# MAPK and Notch-Mediated Effects of Meso-Xanthin F199 Compounds on Proliferative Activity and Apoptosis of Human Melanocytes in Three-Dimensional Culture

**DOI:** 10.1155/2021/8463161

**Published:** 2021-07-20

**Authors:** Irina N. Saburina, Irina M. Zurina, Nastasia V. Kosheleva, Anastasiya A. Gorkun, Elena N. Volkova, Olga S. Grinakovskaya, Anton S. Rybakov, Anna L. Kaysheva, Arthur T. Kopylov, Sergey G. Morozov

**Affiliations:** ^1^Institute of General Pathology and Pathophysiology, 8 Baltiyskaya Str., 125315 Moscow, Russia; ^2^Institute for Regenerative Medicine, Sechenov First Moscow State Medical University, 8-2 Trubetskaya Str., 119991 Moscow, Russia; ^3^World-Class Research Center “Digital Biodesign and Personalized Healthcare”, Sechenov First Moscow State Medical University, 8-2 Trubetskaya Str., Moscow 119991, Russia; ^4^Institute of Biomedical Chemistry, Biobanking Group, 10 Pogodinskaya Str., Bld. 8, 119121 Moscow, Russia

## Abstract

Meso-Xanthin (Meso-Xanthin F199™) is a highly active antiaging injection drug of the latest generation. The main acting compound is fucoxanthin, supplemented with several growth factors, vitamins, and hyaluronic acid. Previous examination of fucoxanthin on melanocytes showed its ability to inhibit skin pigmentation through different signaling pathways focused on suppression of melanogenic-stimulating receptors. In turn, the anticancer property of fucoxanthin is realized through MAPK and PI3K pathways. We aimed to evaluate the effect of fucoxanthin and supplemented growth factors on melanocyte growth and transformation at a proteomic level. The effect of fucoxanthin on melanocytes cultivated in three-dimensional (3D) condition was examined using high-throughput proteomic and system biology approaches to disclose key molecular events of the targeted action. Our results demonstrated significant inhibition of cell differentiation and ubiquitination processes. We found that the negative regulation of *PSME1* and *PTGIS* largely determines the inhibition of NF-*κ*B and MAPK2. Besides, fucoxanthin selectively inhibits cell differentiation via negative regulation of Raf signaling and the upstream activation of IL-1 signaling. It is assumed that inhibition of Raf influences the Notch-4 signaling and switches off the MAPK/MAPK2 cascade. Blockage of MAPK/MAPK2 is feasible due to suppression of Ras and NF-*κ*B by the addressed action of *IKKB*, *IKK2*, and *TRAF6*. Suggestively, Meso-Xanthin F199™ can manage processes of proliferative activity and inhibition of apoptosis due to composition of fucoxanthin and growth-stimulating factors, which may increase the risk of skin cancer development under certain condition.

## 1. Introduction

Meso-Xanthin, commonly known under the trademark Meso-Xanthin F199™, is a highly active antiaging drug of the latest generation, recently developed by the ABG Lab LLC (USA), and recommended to patients under 40 years of age. It consists of several acting compounds, the main of which is fucoxanthin (FX), supplemented with vitamins (A, C, and E), hyaluronic acid, and growth factors (EFG, IGF-2, and *β*EGF). The composition provides a comprehensive effect on problems raised as skin aging. The main compound with the exact antioxidant property, fucoxanthin, is one of the most abundant carotenoids isolated from brown seaweeds and diatoms. Unlike many other carotenoids, the structure of FX is easily affected by heating and UV light exposure [1]. Recent studies demonstrated a wide variety of biological properties of FX promising in treatment and prevention of obesity [2], cancer development [3], and diabetes mellitus [4].

Due to high instability and flexibility of FX under UV irradiation and heating, its effect has been extensively investigated on cells under different conditions. Examination of FX on melanocytes demonstrated the pronounced inhibiting effect on tyrosinase activity and melanin synthesis associated with the decreased cyclooxygenase-2 (*COX2*), endothelin receptor A, and expression of prostaglandin E receptor-1 [5]. It has been proposed that the effect of FX might be accrued through the inhibition of prostaglandin E2 synthesis [5]. In turn, malfunction of *PTGIS* is fraught with a significant decrease in activity and abundance of prostaglandin E2. Since dysregulation of *COX2*, prostaglandin E2, and, consequently, *PTGIS* is tightly associated with the enhanced photosensitivity, there is growing interest in antioxidant and protective properties of FX under UV irradiation.

Food supplements with additives of FX can be efficiently utilized to regulate lipid metabolism [6]. Studies of anticancer action showed that FX mainly arrests cell growth and induces apoptosis [3]. A low dose of FX (under 15 *μ*M) induces cell cycle arrest in the G0/G1 phase and increases the population of cells in the G2/M phase [7]. The antiproliferative property of FX makes this compound a beneficial dietary additive. As there are numerous studies, it has been proposed that antitumor properties of FX are mediated by the upregulation of the ROS-mediated Bcl-xL pathway [8] and downregulation of the JAK/STAT pathway [7] in association with NF-*κ*B. The balance between cell proliferation and cell death is constituted by the importance of the apoptosis process, the disturbance of which generates malignancy. The proapoptotic effect of FX is caused by the ability of ROS generation, which triggers the Bcl-xL pathway [9]. Other research demonstrated that the loss of mitochondrial membrane potential causes the main reason for FX-induced apoptosis, and no concatenated accumulation of ROS was observed [8]. In contrast, some researchers reported on DNA fragmentation following the pretreatment with FX [10]. Eventually, *in vivo* and *in vitro* models evidenced that FX-induced apoptosis is guided by the upregulation of caspase proteins and downregulation of MAPK signaling and Bcl-xL [11]. The NF-*κ*B pathway makes a significant input in the regulation of apoptosis through the binding with Bcl-2 and Bcl-xL promoters; thus, its overexpression endorses the antiapoptotic effect.

To summarize, there is no explicit understanding of the exact mechanisms of antiproliferative and proapoptotic actions of FX. Our research does not intend to explain such complicated events, but the obtained results produce a noticeable sensation that contradicts the majority of known properties of FX. We performed a proteomic assay carried out on melanocyte cell culture grown in a monolayer and as spheroids. The spheroid culture was either exposed to FX or remained untreated, and alterations between conditioned proteomes were evaluated. The main goal of the research was to investigate the net benefit of remedy composition on melanocytes.

Turning to the panoply of evidence-based results in numerous previous investigations, we expected the unambiguous manifestation of antiproliferative effect after exposure to FX. We chose spheroid culture since the monolayer cultured cells are typically characterized by a thin cell body and inappropriate morphology shape of the growing differentiating cells [12]. Therefore, changing the condition to a three-dimensional one was shown to be a more properly adapted model of cell growth and differentiation due to the regular organization of extracellular matrix architecture, cytoskeleton arrangement, and terminal differentiation [13].

Although the obtained results come at odds with the commonly accepted, we observed that FX, combined with growth factors presented in Meso-Xanthin F199™ remedy, significantly inhibits cell differentiation and ubiquitination processes obligatory for differentiation. At the same time, the examined drug notably enhances cell proliferative activity. It has been found that the negative regulation of *PSME1* and decrease in *PTGIS* are mainly determined by the inhibition of IL-1-mediated signaling and by inhibition of NF-*κ*B and MAPK2. Besides, FX is attractive to the significant inhibition of cell differentiation via negative regulation of Raf signaling and the upstream IL-1-mediated signal transduction. In turn, we assume that Raf inhibition influences Notch-4 signaling and the switching to MAPK/MAPK2 signaling. Blockage of MAPK/MAPK2 is available due to indirect inhibition of Ras and NF-*κ*B through the stimulation of their inhibitors found abundantly in our research.

In general, the complicated network managed in melanocytes suggests that FX focuses on the increasing proliferative activity, whereas differentiation and apoptosis endpoints are significantly suppressed. Based on the obtained evidence, we assume that the main targets in Meso-Xanthin preparation are along with the Raf- and IL-1-mediated systems, and the effect is more addressed to *YWHAD*, *PTGIS*, and *PSME1* factors.

## 2. Materials and Methods

### 2.1. Reagents

Trifluoroacetic acid (99%, Reagent Plus®), TEABC (triethylammonium bicarbonate, 1 M solution, pH~8.5), 4-vinylpyridine (95%), and sodium deoxycholic acid (>97% titration) were from Sigma (St. Louis, MO, USA). Acetonitrile (HPLC grade, filtered for 0.2 *μ*m) was purchased from Fisher Chemical (Loughborough, U.K.). Trypsin (sequencing grade modified) was supplied by Promega (Madison, WI, USA). Water (TOC < 3 ppb, up to 18.5 m*Ω*∗cm) was obtained from the Milli-Q Integral 3 purification system, Millipore SAS (France). Urea (99%) and formic acid (98%+, pure) were obtained from Acros Organics (Geel, Belgium). Acetic acid (EMSURE®, glacial and anhydrous for analysis) was from Merck (Darmstadt, Germany). TCEP (tris(2-carboxyethyl) phosphine hydrochloride) was purchased from Pierce™ (Thermo Fisher, Rockford, IL, USA).

### 2.2. Cultivation of Melanocytes

The study was performed using the primary culture of human melanocytes (104-05n, Cell Applications, Inc.). Upon transportation, cells were thawed, resuspended in a complete melanocyte growth medium provided by the manufacturer (135-500, Cell Applications, Inc.), and placed on culture dishes at a density of 10^4^ cells/cm^2^ (monolayer cells designated as M2319). Full growth medium was replaced every two-three days. The culture was passaged upon reaching a 60% confluent state. For the further proteome studies, suspension of melanocytes at passage 4 was used to obtain spheroids under 3D nonadhesive conditions in agarose plates as described previously [14]. Meso-Xanthin was added to the growth medium of experimental spheroids (cells designated as Am02) at the 1 : 10 ratio (normalized to 50 *μ*M of fucoxanthin as the main compound of Meso-Xanthin F199™). The same volume of sodium chloride (NaCl) solution was added to the growth medium in the control group (cells designated as Nm01). Spheroids from experimental and control groups were harvested and collected on day 7 in 1.5 mL plastic tubes, and the growth medium was completely removed by centrifugation in phosphate buffer saline. The resulting pellets of only spheroid cells were frozen at -80°C and stored until sample preparation for the proteomic analysis.

### 2.3. Sample Preparation

Cells (2.5∗10^5^) were resuspended to 200 *μ*L of lysis buffer consisting of 5 M urea, 100 mM TEABC, and 1% deoxycholic acid sodium salt and supplied with 7.5 mM TCEP. Cell suspensions were sonicated on ice for 5 cycles (at 70% power) for 90 seconds per cycle. Following sonication, samples were incubated at 40°C for 30 minutes to perform TCEP-supplied reduction of cysteine residues. The reduced cysteines were alkylated by 2% solution of 4-vinylpyridine in 30% isopropanol for 20 minutes at ambient temperature. Samples were diluted to 1.2 mL finally by 75 mM TEABC and supplied with trypsin (12.5 *μ*L per sample or 1.25 *μ*g). The digestion with trypsin was split into two distinct steps: the first step was carried out at 37°C for 3 hours and the second step was accomplished by the addition of the next trypsin aliquot amount of 1 *μ*g (10 *μ*L per sample) and incubation for the next 2 hours at 42°C. The reaction mixtures were chilled and inhibited by 12 *μ*L of 100% formic acid (1% final concentration). Samples were centrifuged at 10,500 *g* to sediment the insoluble deoxycholic acid, and the obtained supernatants were dried under vacuum at 30°C for 90-120 minutes. The resulting dried pellets were reconstituted in 50 *μ*L of 0.5% formic acid to perform LC-MS analysis.

### 2.4. Liquid Chromatography and Mass Spectrometry

Liquid chromatography was carried out on an Ultimate 3000 RSLC Nano (Thermo Scientific; Waltham, MA, USA) system. Peptides were loaded onto an enrichment column Acclaim Pepmap® (5 mm × 0.3 mm, 300 Å pore size, 5 *μ*m particle size) for 6 minutes at a flow rate of 20 *μ*L/min in water with 3.5% acetonitrile supplemented with 0.1% formic acid and 0.05% acetic acid (mobile phase C, pH = 2.75 ± 0.1 at 20 ± 2°C). After enrichment, peptides were washed out and separated for 90 minutes (including column equilibration time 10 minutes) onto an analytical column Acclaim Pepmap® (75 *μ*m × 150 mm, 1.8 *μ*m particle size, 60 Å pore size) at 0.30 *μ*L/min flow rate in a gradient of mobile phase A (water; pH 2.6 ± 0.15 at *t* = 20 ± 2°C) and mobile phase B (70% acetonitrile and 30% methanol) both supplied with 0.1% formic acid and 0.03% acetic acid.

Detection of peptides was accomplished using a high-resolution benchtop Orbitrap Q-Exactive HF-X (Thermo Scientific) mass spectrometer operated in a positive ionization mode and equipped with a nanoflow NSI ion source. Precursor ions in a range of 420-1200 *m*/*z* were surveyed at a resolution of *R* = 60 K and isolated by quadrupole within ±1.5 Th window with +0.25 Th offset. The maximum integration time for precursor ions was set to 10 ms, or AGC (acquisition gain control) was set to 3*e*6 ions. Tandem scanning was triggered in the top 20 modes, if the signal-to-noise ratio achieved above 1500 counts and precursor ions carried charge from *z* = 2+ to *z* = 6+. Fragment ions were accumulated for a maximum integration time of 60 ms (or AGC was set to 1*e*5 ions), enforced by HCD activation energy (normalized to 27% and ranged within ±20%) and detected in an ultrahigh field orbital mass analyzer at a resolution of *R* = 15 K. The complete duty cycle was estimated for 2.5 sec.

### 2.5. Data Analysis

Data files in RAW format were converted to peak lists using MS Convert (Proteome Wizard). The search was conducted using Search GUI version 3.3.20 (CompOmics). Identification was performed against a concatenated target/decoy database of human proteins (UniProt release August 2020). The decoy sequences were populated by reversing target sequences. Data were processed using the X!Tandem search engine with the following parameters: trypsin as a digestion enzyme with maximum allowed two missed cleavages, resolved charge states varying between *z* = 2+ and *z* = 6+, precursor mass tolerance of ±5 ppm, and fragment ion tolerance of ±0.001 Da. The following variable modifications were chosen: deamidation of Q/E, methionine single oxidation, and 4-hydroxyproline. Fixed modification was pyridilethylation of cysteine residue by 4-vinylpyridine. Results were extracted at no more than 1% of FDR level estimation using decoy hit distribution. Proteomes were treated by the permutation-based test to determine the possible variance between the studied conditions. Bias correction of intra- and intergroup comparisons was evaluated using Spearman's correlation test, and significances of between-group protein frequencies were evaluated by Fisher's exact test with a significance level of *p* < 0.01. Functional analysis was conducted using the Gene Ontology tool with the PANTHER Overrepresentation Test (release 16) with the Bonferroni correction for multiple testing. Human molecular pathways were extracted from the Reactome (version 3.7, database release 75).

## 3. Results

**Figure 1:**
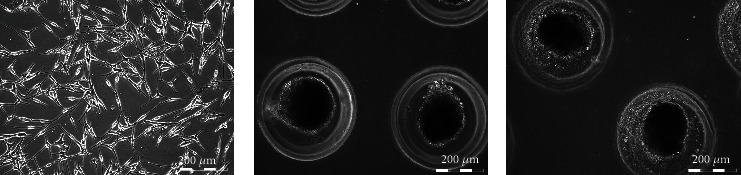
Morphology of melanocytes in 2D and 3D cell cultures. In passage 4, the culture of human melanocytes was represented by cells with spindle-shaped or dendritic cells (a). Cells in nonadhesive microwells formed dark compact spheroids both in control growth medium (b) and in the presence of Meso-Xanthin F199™ (c). Light phase-contrast microscopy.

Melanocytes were cultivated in monolayer (cell M2319) adherent culture and in three-dimensional (spheroids) culture without (cell Nm01) and under exposure to FX (cell Am02). Primary human melanocytes in monolayer culture at passage 4 were spindle-shaped or had a few dendrites (Figure 1(a)). When placed under nonadhesive conditions, cells formed compact spheroids within 7 days both in standard growth medium and in the presence of Meso-Xanthin (Figures 1(b) and 1(c)). Moreover, both control and experimental spheroids became darker at day 7 of cultivation. The difference between groups could not be assessed visually because spheroids were dense. However, previously using spectrophotometric analysis, it has been shown that 50 *μ*M fucoxanthin could significantly downregulate melanin synthesis in spheroids [14].

**Figure 2:**
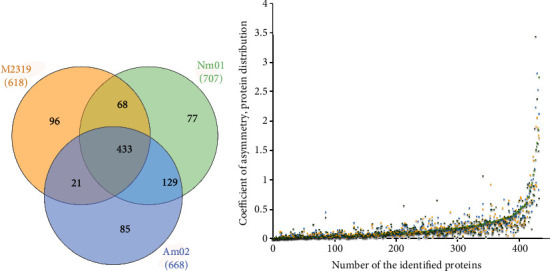
Descriptive primary analysis of the obtained proteomes among studied melanocyte cells cultivated in a monolayer and in spheroids before and after exposure to FX (a). Protein distribution among studied human melanocyte cultures. The total size of the significantly contributing proteome was 434 protein identifications, which is about 19% of the individual proteome of each sample. (b) Distribution of the symmetry coefficient dependent on the cumulative number of protein identifications. The maximum increase in the symmetry coefficient is observed with the accumulation of 80%-87% of the summarized size of the proteome. Most of the identified proteins among studied melanocyte samples make an insignificant contribution to the growth of the asymmetry of the distribution (up to 0.0025 units per protein).

We conducted a proteomic survey of cells upon 7 days of cultivation. After validation and percolation of the acquired proteomes at 1% FDR (false discovery rate), we obtained 2618 different protein identifications attributed to the M2319 cell, 2707 proteins in Nm01 cells, and 2668 different proteins attributed to the proteome of Am02 cells after exposure to FX (Supplementary Materials (available here)). Symmetry comparison of proteomes revealed the group of 2246 commonly identified proteins or 83.78% ± 1.95% of the individual size of proteomes (Figure 2(a)), indicating a moderate heterogeneity among the studied cells, which can be caused by the cooperative influence of FX, growth factors (EFG, IGF-2, and *β*EGF), and cell growth conditions (monolayer and spheroids). After imputation and elimination of redundancy, the normalized contribution of the significantly differed part of the proteome consisted of 434 protein identifications among the studied cell population.

Since the intersection of proteomes is high (85.45% ± 4.55%), one can assume that the affected part of the common proteome belongs to highly abundant proteins. In contrast, the low abundant proteins were less affected by the exposure to FX. The asymmetric distribution of the protein population confirms the suggestion (Figure 2(b)). With the increment of the number of identified proteins in a particular subculture, the coefficient of asymmetry shifts toward a positive direction. It reaches values from 2.5 to 3.8 units, and the trend persists for all subcultures of the study. The maximum rate of the asymmetry coefficient can be traced with the accumulation of at least 380 proteins or at 80%-87% of the total proteome size, while up to this point (Figure 2(b)), the growth of the coefficient is meager and amounts to no more than 0.025 units per protein. Hence, further analysis was performed after renormalization and alignment of the shared proteome between the studied subcultures.

**Figure 3:**
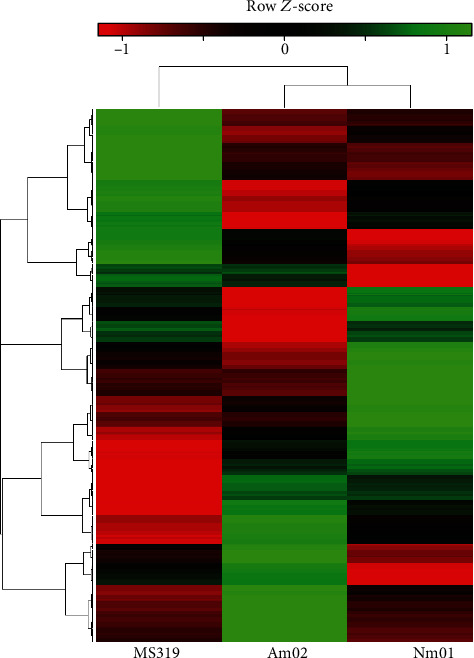
Heatmap of semiquantitative protein distribution in the shared proteome for M2319, Nm01, and Am02 melanocytes. The sample M2319 was employed as a baseline for normalization. Range correlation of proteins was performed using Spearman's test. The total size of the tested proteome involved 434 protein identifications. Samples Nm01 and Am02 are at the closest localization according to the results of cluster analysis, while M2319 is the most distal.

The clustering and Spearman's range correlation analysis were carried out toward the M2319 culture considered the control sample. The study cared fraction of the proteome shared between subcultures (434 proteins) with the significance level of *α* < 0.01. Proteins with the fold changes of FC > 2 or FC < 0.5 at a *p* < 0.01 were accounted as positively or negatively regulated, correspondingly (Figure 3). According to the clustering and semiquantitative analysis, the common proteome elements were distributed as follows: the Nm01 cells were characterized by 61 upregulated and 45 downregulated proteins and the Am02 cells (affected by FX) were comprised of 62 upregulated and 65 downregulated proteins (Figure 3).

The majority of upregulated proteins are involved in the RNA localization and transport (Supplementary Materials). Another large group consisted of proteins regulating the localization on telomeres and the Cajal body (CB) organization, which is in agreement with the RNA transport and localization because the presence of Cajal bodies is a sign of actively undergoing assembling of ribonucleoprotein complexes and processing of small nuclear RNA (snRNA). Functional analysis also deposed proteins toward the binding and stabilization of ribosomal RNA (rRNA binding (GO: 0019843; *р* = 2.15*e* − 06), structural constituent of the ribosome (GO: 0003735; *р* = 8.32*e* − 12), and a cognate group of proteins regulating translation initiating factor (translation initiation factor binding (GO: 0031369; *р* = 2.02*e* − 04)). A more significant specialized group in functional analysis proteins endorses binding and exchange of guanidine bases, heterocycle molecules, and nucleoside phosphates.

**Figure 4:**
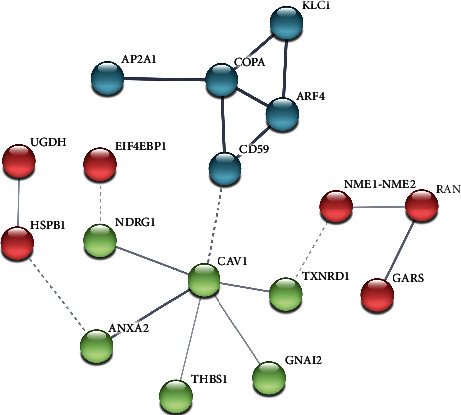
Network interaction analysis of proteins with positive regulation in cells before (Nm01) and after (Am02) exposure to Meso-Xanthin F199. The resulting network establishes interactions in the streamlining processes of cell response to stimulation and activation of RNA metabolism. The background biological processes actively undergoing after exposure to Meso-Xanthin are a response to stimulation (GO: 0051716, *p* = 0.0088), which reflects changing the state and activity of the cell (including gene expression, secretion, and enzymatic activity) and the closely related process of signal transmission (GO: 0007165, *p* = 0.0131). In general, these biological processes agree well with those molecular functions that have been identified in this study (protein binding and protein metabolism and RNA binding and metabolism). In addition, groups of proteins with phosphatase and GTPase activity (GO: 0003924, *p* = 0.0105), which are upstream to protein metabolism actively involved in translation and assembly of ribonucleoproteins, were also identified in this network.

The interaction network analysis revealed an interactome core, organized by the process of cellular response to stimuli (GO: 0051716, *р* = 0.0088; Figure 4). However, this term is a general definition of any process affecting the cell state and activity, including level of gene expression, secretion, and enzymatic activity. The response to stimuli is tightly joined with the signal transduction (GO: 0007165, *р* = 0.0131), defined as the second major functional cluster. In general, these molecular functions correspond well with the biological processes of RNA metabolism, stability, binding, and metabolism of proteins, as has been touched before. Besides, we designated a group of proteins with GTPase and phosphatase activities (GO: 0003924, *р* = 0.0105) involved in the metabolism of proteins and other processes requiring high energy consumption and exchange (i.e., ribonucleoprotein assembly and translation). The more profound analysis of upregulated proteins entails specific reaction pathways, of which the most important was receptor tyrosine kinase-mediated signaling (HSA-9006934, *р* = 0.0308). Notwithstanding, this process encompasses a vast number of different up- and downstream reactions on the scale of numerous interconnected biological processes.

The downregulated group consisted of 65 elements in the Am02 sample and 45 proteins in the Nm01 sample. The network organization is complementary to those for the upregulated proteins (Supplementary Materials). The overall part of the proteome between these two samples was represented by only 22% of the generalized proteome. There were 20 and 32 proteins identified specifically in Nm01 and Am02 samples, correspondingly. Proteins of Am02 arranged the tight network of interactions with a 87% power, which means that most of the proteins are involved in the multifaceted, interconnected biological processes (PPI = 1.0*e* − 06, Figure 4). A closer investigation of the downregulated processes revealed that they functionally replicate those identified in the upregulated groups; however, their biological functions are opposite. A large group of proteins covered the RNA metabolism process (HSA-8953854, *р* = 6.48*е* − 10) but was targeted to the inhibition of RNA hydrolysis and RNA depolymerization, thus empowering the stability of RNA. Hence, despite the fact that these processes involve RNA uptake and processing, they purpose to prevent RNA destruction and guide ribosome stabilization.

## 4. Discussion

### 4.1. Upregulated Group of Proteins

#### 4.1.1. Influence on the RNA Metabolism and Ribonucleoprotein Assembling

To determine alterations in biological processes essentially after the exposure to FX, we enriched the group of upregulated proteins by the stable (constituent) fraction of the proteome, i.e., proteins insignificantly altered before and after being affected by FX. In this context, the amended protein population displayed a few new meaningfully contributing processes of activation and regulation of translation (GO: 0006412, *р* = 9.54*е* − 40) and cotranslational targeted localization of proteins (GO: 0006613, *р* = 9.13*е* − 38). Meanwhile, elongation of peptide chains (HSA-156902, *р* = 7.93*е* − 42) has emerged as the most distinct process coupled with the translation and to be a part of the comprehensive protein metabolism process. Nevertheless, RNA transport/catabolism in combination with nitrogen compound (including nucleotides) metabolism remained the principal process. Both can be easily incorporated into the translation and transcription processes.

Upon closer examination, the main positively regulated processes in the Nm01 and Am02 melanocyte cultures are translation, elongation of the peptide chains, assembly of ribonucleoproteins, and RNA catabolism. In general, actively undergoing synthesis and biogenesis are tightly associated with both stimulation of the cell culture and the gained cell proliferation. The presence of the protein cluster involved in the arrangement of Cajal bodies may indicate a possible malignancy process [15] in Am02 melanocytes after treatment with FX.

We should emphasize that in the Am02 (after exposure to FX) and to a lesser extent in Nm01, we observed a cluster (consisting of 23 proteins) involved in the development of the epithelium (GO: 0060429, *p* = 0.00114), and nearby half of the represented proteins are upregulated. A small fraction of proteins (14 proteins) with a strong clustering factor (PPI = 0.987) traps the proteins associated with morphogenesis and cell differentiation processes. However, this may be due to both the effect of exposure to FX and the regular course of cellular differentiation since this fraction of proteins was distributed evenly among Nm01 and Am02.

#### 4.1.2. Regulation of MAPK4/MAPK6 through the Enhanced Ubiquitin-Related Activity

The most standing out process is found in the Am02 melanocytes against the background related to the Rap1 signaling pathway. The Rap1 family is characterized by small GTPase proteins controlling various sides of cellular activities, including adhesion, polarization, proliferation, and differentiation [16]. Regulation of these activities occurs through the uptake of GDP to GTP and is being accomplished by GEF (guanidine-exchange factors). Several Rap1-related proteins (*GBB1*, *GBG12*, *TSP*, and *ITB1*) with positive regulation (FC varied from 2.73 to 3.18, Table 1) were observed. Considering that *ACTB*, *CDC42*, *HSPB1*, *IQGAP1*, *PSMC2*, *PSMC3*, *PSMC4*, *PSMD2*, *RAP1B*, and *VCL* were established among the upregulated cohort after exposure to FX, it seems that the Rap1 signaling pathway further excites the MAPK cascade (*p* = 0.0057), which eventually leads to cell proliferation. Some of these proteins belong to the MAPK4/MAPK6 branch (*p* = 0.0031), while others were assigned to the MAPK2 signaling pathway (*p* = 0.0064). Both MAPK6 and MAPK4 kinases, also known as ERK3 and ERK4, are atypical kinases. The observed elevation of MAPK4 abundance (FC = 1.92, Table 1) is in agreement with the recent finding of the strong association of MAPK4 overexpression with the rapid tumor development and metastasis via phosphorylation of *AKT* and downstream activation of the PI3K/AKT/mTOR pathway that regulates cell surveillance [17]. The MAPK6 enzyme also differs from other family members: it is an unstable kinase, whose uptake largely depends on the rate of ubiquitin-dependent degradation [18]. Little is known about this signaling pathway, apart from coactivation of MAPK6 by *CDC42* [18], which has been observed in our study in all melanocyte subcultures, but the level change of *CDC42* between conditions was insignificant.

Overexpression of MAPK4 leads to the activation of its known substrate, mitogen-activated protein kinase-activated protein kinase 5 (*MK5*), which is a marker of tumor onset at a very early stage of development, and initiates cell migration [19]. In this respect, gradual increase in cell viability due to inhibition of apoptosis can be expected. There are a broad number of direct and indirect regulators of apoptosis mechanisms, but Bcl-2 is, probably, the most scrutinized one. The very fact that overexpression of Bcl-2 delays the apoptosis makes this protein the crucial guider in the selection of long-living cells. However, data regarding the prevalence and the expression level of Bcl-2 in cancer cells are controversial. Although most cancer cases are characterized by the increased level of Bcl-2, some authors reported on low expression of Bcl-2 in breast cancer and Waldenström macroglobulinemia [20, 21]. Typically, the elevated activity of Bcl-2 family proteins is indicated in the primary malignant cells, while later the regulation of apoptosis may occur in an MAPK-independent manner; thus, the level of Bcl-2 remained almost unaltered or slightly decreased [22]. In this study, we observed moderately increased expression level of Bcl-2 (FC = 1.78, Table 1), which might indicate the very early onset of possible malignancy and cell migration, which is also partially confirmed by the observed lowering of IL-1 release activators discussed below.

The most attractive circumstance is that the only well-known and reliable substrate for both MAPK4 and MAPK6 is the *HSBP1* protein [23, 24]. The *HSBP1* is overrepresented in Am02 culture, and its relative abundance significantly increased after exposure to FX (FC = 2.34, Table 1 and Supplementary Materials). Activation of *HSBP1* in association with MAPK4/MAPK6-mediated cascade entails the rearrangement of cytoskeletal actin widely represented in the studied samples. Furthermore, some of actins (*ARPC2* and *ARPC3*) changed significantly towards the increasing abundance (for example, in sample Nm01 FC = 4.27 and FC = 2.70, respectively, and FC = 2.71 for *ARPC2* in M2319 cells; Table 1). In contrast, other proteins involved in the organization of cytoskeleton architecture decreased in response to stimulation (*DCTN1* to FC = 0.20 and *SRC8* to FC = 0.33), whereas the abundance of filament regulators (*CAPZB* and *CAZA1*) did not vary significantly in either of the studied samples, although these two proteins determine the morphology of cellular cytoskeleton through the Ca^2+^-dependent limiting of polymerization growth [25, 26].

**Figure 5:**
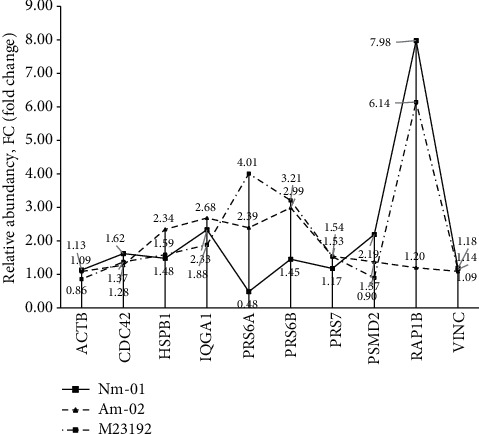
Dynamic alterations of Rap1- and MAPK4/MAPK6-related proteins overlapped between analyzed melanocytes. Proteins regulating the activity of MAPK4 and MAPK6 kinases (*PRS6A*, *PRS6B*, *PRS7*, and *PSMD2*) are characterized by the maximum abundancy in the Am02 culture (after exposure to FX) and significantly decreased in Nm01 melanocytes. The RAP1B protein switching between different signaling cascades takes the most abundance in samples Nm01 and M2319 and is strongly inhibited in Am02 melanocytes after exposure to FX.

Due to regulation of MAPK4/MAPK6 kinases, activities occur in the ubiquitin-dependent manner; the detection of various proteasome regulatory factors attracted a noticeable attention. We analyzed dynamic distribution of MAPK4/MAPK6 regulatory proteins and the main substrates of the pathway's endpoints. Cells before (Nm01) and after (Am02) exposure to FX differed by fractional contribution of proteins controlling the active cell proliferation (Figure 5). It has been found that Am02 subculture is featured by the augmented abundancy of proteasome regulating proteins (*PRS6A*, *PSMC4*, *PSMC2*, and *PSMD2*; Figure 5 and Table 1). At the same time, these proteins were also costarred by a great relative contribution in the M2319 cells (control sample of melanocytes).

#### 4.1.3. Intersection of the Suppressed Ras-Mediated Signaling and Enhanced MAPK4/MAPK6 Pathway in Guiding Melanocyte Proliferation and Differentiation

The most striking peak corresponds to the *RAP1B* or Ras-related protein Rap-1b (Figure 5). Its relative abundance reaches a maximum in Nm01 (FC = 7.98) and M2319 (FC = 6.14) but does not differ in Am02 compared to the control cells. This protein is an important participant of the Ras/Rab signaling pathway and belongs to a large family of proteins switching to and regulating other distinct signaling cascades [27, 28]. The *RAP1B* is strongly inhibited in Am02, and given that the Rap1 signaling has several possible scenarios, it becomes obvious that the effect of FX is focused specifically on the increased cell proliferation and surveillance but, apparently, the effect is mediated through the MAPK4/MAPK6 pathway as has been discussed above. Unlike FX-untreated cells, Am02 switching to cell differentiation and adhesion is extremely complicated since the related network regulators are inhibited. Hence, it is suggested that the main effect of FX on melanocytes is addressed to the Ras/Rab protein family, represented by a large group of proteins with explicit GTPase activity (hsa04014 index in the Reactome). On most occasions, activation of Ras-related proteins occurs in response to the stimulation of extracellular receptors for various growth factors [28], including those accommodated in the Meso-Xanthin F199™ (EFG, IGF-2, and *β*EGF). Having gained a certain stage of signal transduction, they regulate further signaling cascade branching either along with the P3IK/Akt signal, along the MAPK, or along the Rap1 pathway [29], which are parts of the most conservative AMPK transmission (AMP-mediated (activated) kinases).

#### 4.1.4. Enhanced Cell Proliferation Mediated by Notch and Wnt Pathways after Exposure to FX

The clustering of a large number of proteins into Notch (HSA-157118, *p* = 0.0201) and Wnt signaling pathways (HSA-195721, *p* = 0.0114) is an exciting observation after exposure to FX. The Notch pathway greatly drives cell cycle and proliferation; therefore, different Notch-related targets maintain the balance between proliferation and apoptosis. Notch activation is broadly reported as oncogenic, and only a limited number of studies indicated it as a tumor suppressor, for example, in hepatocellular carcinoma and skin cancer [30]. At the same time, Notch signaling is permanently stimulated in continuously renewed tissues such as the skin, blood, or intestinal epithelium [31]. Another pronounced signaling pathway, Wnt, is also extremely important and conservative for cell proliferation, but in addition, it is directly involved in the maintenance of stem cell homeostasis, as well as in the maturation and polarization of cells [32]. In this study, we identified a shell of 210 proteins directly or via secondary interaction involved in both Notch and Wnt signaling pathways, of them *YWHAZ*, *PSMD2*, *PRS6A*, *PRS6B, PRS7*, *ACTA*, *AP2A1*, *CAV1*, *GBG12*, and *STAT1* (Supplementary Materials and Table 1) were the most altered. It should be mentioned that some of these proteins are also tracked for the Rap1 signaling, which leads to the active proliferation and cell polarization in the case of Am02 cells and prohibits other functional branches due to the inhibition of Ras proteins.

In this study, we have observed the high-level expression of Notch-1 receptor (FC = 2.13) and Jagged-1 ligand (FC = 2.98) in melanocytes after exposure to FX (Table 1). Considering that Notch-1 and Notch-2 receptors are typically overexpressed in solid tumors [33], while the inhibition of Notch-1 by the *γ*-secretase complex, oppositely, results in apoptosis [34], one can assume that this event is a starting point in the enhanced proliferative activity. However, evidence exists that overexpression of Notch-2 receptor prevents metastasis and tumor growth [35] and can induce apoptosis making it an attractive target for cancer therapy [36]. In contrast, the constitutive expression of Notch-1 and silencing of Notch-1 by siRNA result in the decrease in proliferative activity and enhance apoptosis [33, 34]. In this study, we did not recognize Notch-2, but the summarized data display a close connection between Notch, Wnt, and Rap-1 pathways in melanocytes after exposure to FX. Together, these pathways are accumulated to enforce cell proliferation and maturation, rather than differentiation of melanocytes. Biogenesis of organelles, protein metabolism, and RNA metabolism, which includes the assembly of ribosomes and the processing of snRNAs, are also vivid processes manifested in Am02 and to a lesser extent in Nm01 cells. Remarkably, the activation of proliferation due to Wnt/Notch signaling is distinctly manifested in Am02. Likewise, the abundances of *AP2A1*, *CAV1*, *YWHAZ*, *GBG12*, and *STAT1* exceeded FC = 2.39 or even more in Am02 compared to M2319 and Nm01 cells. These proteins are well defined to participate in the lateral inhibition, when individual cells within a margin are stochastically selected to accept specific functional specializations, and the Notch pathway prohibits their immediate neighbors from going in the same way [37]. In case of exposure to FX, we can assume substantial progress of the lateral inhibition, which does not permit melanocytes to differentiate but instead orders their continual proliferation.

The assembly of Notch receptors requires reasonably extensive processing and posttranslational modification. The *POGLUT1*-mediated bearing of glycosyl moiety is the main type of modification for pre-Notch receptors [38]. Despite the fact that we did not detect *POGLUT1* in our study, we recognized a high level of *UGDH* (Table 1), which is essential for the release of activated sugars (UDP-sugar) and their transport for the subsequent binding to other enzymes. This protein is implicated in several stages of embryonic development, including synthesis of glycosaminoglycans. Particularly, this protein styles a tight functional complex with *POGLUT1* and is obligatory for the glycosylation of Notch receptors. In Am02 cells, the *UGDH* reaches the maximum relative abundance (FC = 5.54), which may indicate a poorly controlled cell proliferation and maintain cooperatively ongoing biogenesis and metabolism of RNA. Meanwhile, the abundance of *UGDH* in Nm01 (before exposure to FX), on the contrary, was substantially lower and made only FC = 0.4.

### 4.2. Downregulated Group of Proteins

#### 4.2.1. Protection of RNA Metabolism from Hydrolysis and Ribonucleoprotein Dissociation

A comprehensive examination of downregulated proteins uncovered their reiteration to the opposing biological processes relatively to those indicated in the group of upregulated proteins. The bulky cluster was designated to the RNA metabolism (HSA-8953854, *p* = 6.48*e* − 10), but the main difference is concluded in the course of activity. The considered batch of proteins (*DDX39A*, *HNRNPH2*, *HNRNPK*, *HNRNPL*, *HNRNPU*, *PSMA7*, *PSMB1*, *PSMC5*, *PSME1*, and *YWHAB*), in overwhelming majority, intended to inhibit RNA hydrolysis and the process of RNA-protein complex dissociation. Thus, in the context of their molecular functions, the main property of these proteins is focused on the stabilization of RNA and its complexes, including ribosomes.

Other groups of proteins enroll signaling pathways involved in apoptosis (*PLEC*, *PSMA7*, *PSMB1*, *PSMC5*, *PSME1*, and *YWHAB*, *p* = 0.00012); however, their molecular functions control the inhibition of apoptosis, in particular, the abolishment of the proteasome machinery operation, blockage of the phosphorylated (activated) *SRPK2* protein translocation, antagonization of the effect of cyclin D1, and stabilization of cytoskeleton [39].

#### 4.2.2. Inhibition of Apoptosis through Regulation of IL-1 Signaling Mediated by PTGIS and PSME1

**Figure 6:**
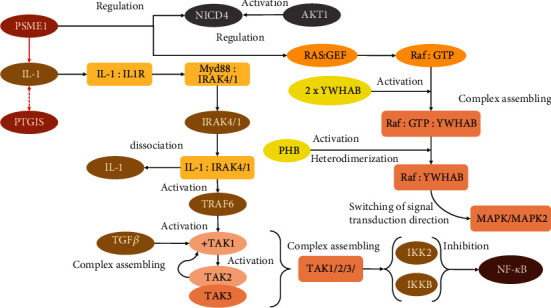
Comprehensive interaction of plenty proteins in signaling pathways inhibited by FX exposure. The protein *PSME1* is one of the main regulators of IL-1, in response to which, in turn, the expression of *PTGIS* is enhanced. The interaction of IL-1 with its single receptor IL1R causes the dissociation of IRAK1 and IRAK4 from the complex with Myd88 and the assembling of a molecular complex with IL-1. This complex, in turn, activates TRAF6, which starts the process of its autoubiquitination. Being in such modified form, TRAF6 interacts with TAK1, which has to be already activated by the TGF-*β* factor. After interaction and activation, TAK1 is bound to a complex of TAK2 and TAK3 subunits and such molecular machinery enhances the expression of the NF-*κ*B inhibitors, like *IKK2* and *IKKB*. On the other hand, *PSME1* is a regulator of the Raf-MAPK pathway where IL-1 interacts with Ras surface receptors and, thus, activates its cytoplasmic GEF domain. Activated RAF with GTP can interfere with the *YWHAB* dimer, and the *PHB* factor initiates their rearrangement and further heterodimerization, which is the starting point for triggering the MAPK/MAPK2 signaling pathway.

Despite the fact that some proteasome subunits are overrepresented in both up- and downregulated groups, their functional significance is fundamentally different. In the group with positive regulation, such proteins were amended for the correction and regulation of the MAPK4/MAPK6 signaling pathway, whereas the group with negative regulation purposed the ubiquitin activation, translocation, and maintenance of degradation systems, i.e., elements constituting the protein utilization (protein metabolism). Their relative abundances are strongly inhibited, meaning the prevalence of stoichiometric advantage of protein biogenesis (anabolic processes), which makes *PSME1* and *PTGIS* of essential interest (Figure 6).

Despite the fact that *PSME1* and *PTGIS* are not functionally interconnected, they have many shared points of intersection and endpoints through various signaling cascades: inhibition of apoptosis, regulation of transcription, and immune response (in particular, during the organization and secretion of MHC-I complexes). Both proteins are signal transducers for the IL-1 ([40]; Figure 6). Likewise, the expression of *PTGIS* is typically affected in response to activation or, conversely, inhibition of IL-1 depending on circumstances [41]. In this study, the abundance of *PTGIS* exhibited diminishing tendency in Nm01 cells (FC = 0.66) and continued to decrease after exposure to FX in Am02 (FC = 0.36). In this respect, we assumed a strong inhibition of IL-1-mediated signaling in melanocytes especially after exposure to FX. Another transmitter, *PSME1*, exactly repeated the abundancy trace of *PTGIS* and continually decreased from FC = 0.38 (in Nm01) to FC = 0.12 (in Am02). The relation of these proteins to IL-1 signaling is widely reviewed in the context of apoptosis inhibition. In particular, IL-1 binds to the IL1R receptor; thereafter, it deploys assembling of the complexes with *IRAK1* and *IRAK4* dissociated from the MyD88 factor before [42].

The formation of the tight complex between receptor-activated IL-1 and IRAK factors enables binding with *TRAF6* if mounted with its receptor. Since *TRAF6* is an E3-ubiquitin ligase, its activation endorses autoubiquitination through the E2-conjugating complex Ubc13/Uev1a [43]. This stands an important and critical point, because the in-complex *TRAF6* can bind with TGF-*β*-activated *TAK1* [44, 45] and facilitate the assembly of the supramolecular complex with *TAK2* and *TAK3* factors, both of which contain zinc finger motifs (Figure 6). The organization of such supramolecular complex causes steric and conformational rearrangements in *TAK1*, thus exhibiting strong activating properties toward *IKK2* and *IKKB* inhibitors of NF-*κ*B kinase (Figure 6). It is important to remind that proteins belonging to the IL-1 signaling pathway (HSA-9020702, *p* = 0.00013) are the most suppressed in our study, which is consistent with ultimately enhanced proliferative activity and dynamic rearrangement of the extracellular matrix and cytoskeleton. The obtained data support our assumption that FX carries a significant inhibition of cell differentiation and ubiquitination processes, which are mandatory for maturation and rearrangement of some organelles during differentiation (for example, during maturation and fusion of mitochondria). In turn, in the context of inhibited IL-1 and NF-*κ*B branches, the network of negatively regulated proteins is consistent with the widely enhanced cell proliferation, biogenesis, and RNA metabolism processes highlighted among positively regulated proteins.

#### 4.2.3. A Complex Vignette of MAPK2, NF-*κ*B, and Notch-4 Interaction as a Part of Large Raf Signaling

The presence of MAPK/MAPK2-related proteins as downregulated proteins encourages the proposed inhibiting effect of FX. Unlike MAPK4/MAPK6, this branch is concerned with more general purposes and triggers other pathways, which are necessary to increase expression of various transcription factors working in cooperation with NF-*κ*B and specifying the focal adhesion and differentiation. However, as evidenced above, cell differentiation is under massive suppression due to significant downregulation of MAPK/MAPK2 (FC = 0.22 for *MAPKAPK2*; Table 1) signaling and might be accomplished through the binding of activated *TAK1* factor to *IKK2*, which must be in a molecular complex with the NEMO-modulating protein [46]. That event may play a central role in the regulation of the cell cycle, since suppression of the MAPK2-mediated pathway substantially decreases the frequency of the apoptosis event and intensifies resistance of the tumor to anticancer therapy [47].

The formation of this complex, consequently, may increase the degree of *IRAK* phosphorylation, causing its polyubiquitination. In turn, *IKKB* induces phosphorylation of NF-*κ*B and its release from the complex with the *TPL2* factor making the trigger of the MAPK/MAPK2 signaling cascade impossible [48] (Figures 4 and 6). However, the obtained results suggest that all pathways upstream of MAPK are strongly inhibited, which abrogates the scenario through the activation of MAPK transmission. For this reason, we observed that the majority of MAPK signaling participants are underrepresented (*PEA15*, *PHB*, *PSMA7*, *PSMB1*, *PSMC5*, *PSME1*, *SEPT7*, and *YWHAB*; Table 1 and Supplementary Materials). The dimerization of the downregulated *YWHAB* in Am02 cells is a turning point of wide RAF signaling deployment that determines switching of Ras branch to MAPK/MAPK2. Another significantly suppressed protein (*PHB*, FC = 0.30) is important in lieu of the necessity for the complex organization between activated RAF-GTP and dimerized *YWHAB*. A significant decrease in *PHB* after exposure to FX supplements the suggestion about the largely inhibiting effect of FX toward MAPK/MAPK2 signaling. Besides, it seems that such targeted effect can be accomplished upon binding of the activated complex to *YWHAB* dimers and its further transformation to heterodimer structure (Figure 6).

In differentiating cells, initiation of apoptosis is broadly determined by the Notch-4 factor. It is known that Notch-4 is deeply implicated in cancer development and can be a clinically significant marker of melanoma stem cell function [49]. Despite the fact that the Notch-4 pathway still is ambiguous, it is well investigated that following the phosphorylation by Akt1, the complex of NICD4 with *YWHAZ* causes the Notch-4 intracellular domain (NICD4 domain) to linger in the cytoplasm [50]. Details of Nocth-4 regulation are uncertain, but it determines the initiation of cell differentiation mainly in routes of angiogenesis or neurogenesis [51]. In renal carcinoma tissue, the level of Notch-4 is significantly decreased or almost absent, though it inversely correlates with tumor progression [52, 53]. In breast cancer patients with low level of Notch-4, the overall surveillance is higher (81.8% at 2 years after treatment) compared to patients with high level of Notch-4 (63.2%) [54]. Constitutive expression of Notch-4 in melanoma induces the transition to epithelial-like morphology cells with highly reduced migratory and proliferative activity [55]; thus, decrease in *NOTCH4* expression promotes tumor progression and forces metastasis. We have detected the compact group of downregulated proteins after exposure to FX (*PSMA7*, *PSMB1*, *PSMC5*, *PSME1*, and *SKP1*; *p* = 1.19*е* − 05) that carry the negative impact on Notch-4- (FC = 0.18; Table 1) mediated pathways, thus contributing to the uncontrolled proliferative activity.

In summary, we have to notify that the negative regulation of Notch-4 and IL-1 is fraught with the inhibition of the RAF-MAPK cascade. The latter triggers activation and regulation of gene expression liable for cell differentiation including, but not limited, rearrangement of the cytoskeleton, extracellular matrix architecture, translocation of nuclear proteins, ubiquitination, and reorganization of certain organelles. The RAF-MAPK signaling pathway is initiated in response to binding of various growth factors with corresponding receptors, thence stimulating the downstream activation of RAS and, eventually, launching the MAPK cascade. The signaling pathway belongs to FLT3-mediated cytokine cascades, and the stimulation can be carried out by EGFR or IGF or by interleukins IL-1 and IL-2.

We found that the negative regulation of *PSME1* and the decrease in *PTGIS* largely determine the inhibition of IL-1-mediated signaling. That leads to further inhibition of NF-*κ*B and MAPK2, specifically after exposure to FX. However, it seems that the established points were merely intermediates in the signal transduction order. As far as it has been disclosed, the major intermediate set point is located in the segment of the RAF cascade, guiding the switch between MAPK, IL-1, and NF-*κ*B signaling paths.

## 5. Conclusion

The analysis of the melanocyte proteome displayed contemporaneous activation and inhibition of cognate biological processes after exposure to FX. The processes are running through the upregulation of RNA metabolism and activated translation, peptide chain elongation, assembly of ribonucleoproteins, and RNA catabolism. Turning to the panoply of evidence-based matters, one can suggest that these processes are associated with the enhanced proliferation, stimulated with the FX and supplemented growth factors. Another notable arrangement after exposure to FX (Am02 cells) is placed in the cluster of Cajal body organization, which is a sign of snRNA processing and metabolism and an indicator of possible malignancy.

The activation of Rap1 signaling that controls various cellular processes (cell adhesion, cell polarization, etc.) is one of the most engaged among upregulated proteins. The majority of Rap1 elements intercept with the MAPK4/MAPK6 signaling, where MAPK6 activity is under the control of the ubiquitin-dependent machinery. In this regard, it is fraught with the increased abundance of ubiquitin-related proteins, as well as with some substrates of MAPK4/MAPK6 (particularly, *CDC42* and *HSPB1*).

Some proteins are distributed between Notch and Wnt pathways; however, insofar melanocytes belong to continually renewed cells, and engaging the Notch in permanent activity does not produce a significant sensation. In turn, Wnt is the major way in maintaining stem cell homeostasis and their proliferation. A large fraction of Wnt- and Notch-related proteins are shared with Rap1 signaling, having placed the affected melanocytes to enhanced proliferative and polarization activity and abolishing other branches of development due to the inhibition of Ras proteins.

The environment of negatively regulated proteins looks different, but in general, it is complementary by molecular functions. Structural integrity of RNA and ribonucleoprotein complexes through the inhibition of RNA hydrolysis and catabolism of protein molecules is the major process under focus.

Besides, inhibition of apoptosis, stabilization of cytoskeleton, and protein utilization are also parts of protein metabolism. FX selectivity is primarily attentive on the significant inhibition of cell differentiation and their specialization. The RAF signaling and its upstream activation through IL-1 are negatively regulated. In turn, RAF retards the influence on Notch-4 signaling and on the switching to MAPK/MAPK2 signaling, thus closing cellular differentiation. Blockage of MAPK/MAPK2 becomes available due to indirect inhibition of Ras and NF-*κ*B through the stimulation of their inhibitors (*IKKB*, *IKK2*, and *TRAF6*).

In summary, the managed network of molecular events suggests that FX focuses on the increasing proliferative activity and cell maturation, whereas differentiation is significantly suppressed. Based on the obtained evidence, we assume that the main target of FX are the Raf- and IL-1-mediated system, and more addressed to *YWHAD*, *PTGIS* are *PSME1* elements.

## Figures and Tables

**Table 1:** The most significantly differed proteins exhibited in the primary culture of human melanocytes cultured under three-dimensional condition (spheroids) for 7 days and exposed to 50 *μ*M equivalent of FX (Am02) or treated with saline solution (Nm01).

Main accession	Recommended protein name	Gene	Ensembl gene ID	Nm-01, FC	Am-02, FC	*p* value
Q06323	Proteasome activator complex subunit 1	PSME1	ENSG00000092010	0.38	0.12	6.05*E* − 04
Q99466	Neurogenic locus notch homolog protein 4	NOTCH4	ENSG00000204301	1.17	0.18	2.59*E* − 04
Q14203	Dynactin subunit 1	DCTN1	ENSG00000204843	0.64	0.20	3.98*E* − 05
P49137	MAP kinase-activated protein kinase 2	MAPKAPK2	ENSG00000162889	1.12	0.22	3.62*E* − 04
Q16181	Septin-7	07-Sep		0.75	0.29	5.50*E* − 05
P35232	Prohibitin	PHB	ENSG00000167085	0.60	0.30	1.38*E* − 04
P55795	Heterogeneous nuclear ribonucleoprotein H2	HNRH2	ENSG00000126945	0.37	0.31	5.10*E* − 04
Q15121	Astrocytic phosphoprotein PEA-15	PEA15	ENSG00000162734	0.29	0.32	1.34*E* − 04
Q14247	Src substrate cortactin	SRC8	ENSG00000085733	1.62	0.33	1.71*E* − 04
Q16647	Prostacyclin synthase	PTGIS	ENSG00000124212	0.66	0.36	3.17*E* − 05
Q15149	Plectin	PLEC	ENSG00000178209	0.20	0.40	1.25*E* − 04
P63208	S-phase kinase-associated protein 1	SKP1	ENSG00000113558	0.40	0.40	6.57*E* − 04
P61978	Heterogeneous nuclear ribonucleoprotein K	HNRPK	ENSG00000165119	0.64	0.41	3.28*E* − 05
P14866	Heterogeneous nuclear ribonucleoprotein L	HNRPL	ENSG00000282947	0.84	0.43	3.34*E* − 04
P31946	14-3-3 protein beta/alpha	1433B	ENSG00000166913	0.71	0.50	4.11*E* − 04
Q00839	Heterogeneous nuclear ribonucleoprotein U	HNRPU	ENSG00000153187	0.91	0.50	1.45*E* − 04
Q92499	ATP-dependent RNA helicase DDX1	DDX1	ENSG00000079785	5.08	0.60	3.52*E* − 05
Q99623	Prohibitin-2	PHB2	ENSG00000215021	0.42	0.63	2.75*E* − 05
O15145	Actin-related protein 2/3 complex subunit 3	ARPC3	ENSG00000111229	2.70	1.03	2.05*E* − 04
O15144	Actin-related protein 2/3 complex subunit 2	ARPC2	ENSG00000163466	4.27	1.20	2.36*E* − 04
P61224	Ras-related protein Rap-1b	RAP1B	ENSG00000127314	7.98	1.20	2.00*E* − 04
Q13200	26S proteasome non-ATPase regulatory subunit 2	PSMD2	ENSG00000175166	2.19	1.37	2.46*E* − 04
P52272	Heterogeneous nuclear ribonucleoprotein M	HNRPM	ENSG00000099783	2.62	1.45	9.07*E* − 05
Q04917	14-3-3 protein eta	1433F	ENSG00000128245	2.65	1.53	1.14*E* − 04
P10415	Apoptosis regulator Bcl-2	BCL2	ENSG00000171791	0.73	1.78	3.69*E* − 03
P40121	Macrophage-capping protein	CAPG	ENSG00000042493	2.32	1.79	8.15*E* − 05
P42224	Signal transducer and activator of transcription 1-alpha/beta	STAT1	ENSG00000115415	1.32	1.84	1.10*E* − 04
P31152	Mitogen-activated protein kinase 4	MAPK4	ENSG00000141639	0.68	1.92	3.38*E* − 05
P46531	Neurogenic locus notch homolog protein 1	NOTCH1	ENSG00000148400	0.66	2.13	6.71*E* − 04
Q15019	Septin-2	SEPT2		1.05	2.15	7.20*E* − 05
P31943	Heterogeneous nuclear ribonucleoprotein H	HNRH1	ENSG00000169045	0.68	2.21	4.35*E* − 05
P04792	Heat shock protein beta-1	HSPB1	ENSG00000106211	1.48	2.34	5.16*E* − 04
P17980	26S proteasome regulatory subunit 6A	PRS6A	ENSG00000165916	0.48	2.39	4.92*E* − 05
P08670	Vimentin	VIME	ENSG00000026025	0.98	2.61	3.29*E* − 04
P46940	Ras GTPase-activating-like protein IQGAP1	IQGA1	ENSG00000140575	2.33	2.68	2.60*E* − 04
Q9UBI6	Guanine nucleotide-binding protein G/G/G subunit gamma-12	GBG12	ENSG00000172380	1.29	2.73	2.36*E* − 04
P78504	Protein jagged-1	JAG1	ENSG00000101384	0.97	2.98	4.27*E* − 05
P43686	26S proteasome regulatory subunit 6B	PRS6B	ENSG00000281221	1.45	2.99	2.70*E* − 04
Q6NZI2	Caveolae-associated protein 1	CAVN1	ENSG00000177469	1.82	3.56	2.95*E* − 04
O95782	AP-2 complex subunit alpha-1	AP2A1	ENSG00000196961	5.14	3.59	1.78*E* − 04
O60701	UDP-glucose 6-dehydrogenase	UGDH	ENSG00000109814	0.40	5.54	3.40*E* − 05
P60953	Cell division control protein 42 homolog	CDC42	ENSG00000070831	2.14	5.78	9.38*E* − 05
Q03135	Caveolin-1	CAV1	ENSG00000105974	5.32	6.18	7.20*E* − 05
P07996	Thrombospondin-1	TSP1	ENSG00000137801	3.95	6.58	1.34*E* − 04
P62873	Guanine nucleotide-binding protein G/G/G subunit beta-1	GBB1	ENSG00000078369	1.30	20.80	3.15*E* − 04

## Data Availability

Supplementary material is available in the Mendeley Data Repository using the following link: doi:10.17632/nmrfyymrrn.1. Data are represented as a complete proteome shared between assayed melanocytes cultured in a monolayer (M2319) and in spheroids before (Nm01) and after (Am02) exposure to Meso-Xanthin F199™. Data content comprises assigned gene attribution and protein characterization according to the obtained proteomic analyses and table of semiquantitative distribution of the defined proteins (green color indicates the most abundant; red color indicates the least abundant proteins among analyzed cell cultures; semiquantitative data are estimated in linear and logarithmic scales).

## Abbreviations

AP2A1: AP-2 complex subunit alpha-1
ARPC2: Actin-related protein 2/3 complex subunit 2
ARPC3: Actin-related protein 2/3 complex subunit 3
BCL2: B-cell lymphoma 2 proteins; apoptosis regulator Bcl-2
CAPZB: F-actin-capping protein subunit beta
CAV1: Caveolin-1
CAZA1: F-actin-capping protein subunit alpha-1
CB: Cajal body
COX2: Cyclooxygenase-2
DCNT1: Dynactin subunit 1
DDX39A: ATP-dependent RNA helicase DDX39A
EGF: Epidermal growth factor
FC: Fold change
FX: Fucoxanthin
GBB1: Guanine nucleotide-binding protein G(I)/G(S)/G(T) subunit beta-1
GBG12: Guanine nucleotide-binding protein G(I)/G(S)/G(O) subunit gamma-12
HNRNPH2: Heterogeneous nuclear ribonucleoprotein H2
HNRNPK: Heterogeneous nuclear ribonucleoprotein K
HNRNPL: Heterogeneous nuclear ribonucleoprotein L
HNRNPU: Heterogeneous nuclear ribonucleoprotein U
HSPB1: Heat shock protein beta-1
IGF-2: Insulin-like growth factor 2
IKK2: Inhibitor kappa-B kinase 2
IKKB: Inhibitor of nuclear factor kappa-B kinase subunit-beta
IQGAP1: Ras GTPase-activating-like protein IQGAP1
ITB1: Integrin beta-1
JAG1: Protein jagged-1
MAPK: Mitogen-activated protein kinase
MAPK4: Mitogen-activated protein kinase 4
MAPKAPK2: MAP kinase-activated protein kinase 2
MK5: Mitogen-activated protein kinase-activated protein kinase 5
NOTCH1: Neurogenic locus notch homolog protein 1
NF-*κ*B: Nuclear factor-*κ*B
PEA15: Astrocytic phosphoprotein PEA-15
PHB: Prohibitin
PLEC: Plectin
POGLUT1: Protein O-glucosyltransferase 1
PPI: Protein-protein interaction
PRS6A: 26S proteasome regulatory subunit 6A homolog B
PSMC2: 26S proteasome regulatory subunit 7
PSMC3: 26S proteasome regulatory subunit 6A
PSMC4: 26S proteasome regulatory subunit 6B
PSMC5: 26S proteasome regulatory subunit 8
PSMD2: 26S proteasome non-ATPase regulatory subunit 2
PSME1: Proteasome activator subunit 1
PTGIS: Prostaglandin I2 synthase
RAP1B: Ras-related protein Rap-1b
ROS: Reactive oxygen species
rRNA: Ribosomal RNA
SEPT7: Septin-7
SKP1: S-phase kinase-associated protein 1
snRNA: Small nuclear RNA
SRPK2: SRSF protein kinase 2
STAT1: Signal transducer and activator of transcription 1-alpha/beta
TCEP: Tris(2-carboxyethyl) phosphine hydrochloride
TEABC: Triethylammonium bicarbonate
TRAF6: TNF receptor-associated factor 6
TSP: Thrombospondin-1
UGDH: UDP-glucose 6-dehydrogenase
UV: Ultraviolet
VCL: Vinculin
YWHAB: 14-3-3 protein beta/alpha
YWHAZ: 14-3-3 protein zeta/delta.

## Supplementary Materials

Supplementary Materials Data content comprises assigned gene attribution and protein characterization according to the obtained proteomic analyses and table of semiquantitative distribution of the defined proteins (green color indicates the most abundant; red color indicates the least abundant proteins among analyzed cell cultures; semiquantitative data are estimated in linear and logarithmic scales). Data are deposited in the Mendeley Data Repository and available at the following link: doi:10.17632/nmrfyymrrn.2.
