# Supplementary material for: MAPK and Notch-Mediated Effects of Meso-Xanthin F199 Compounds on Proliferative Activity and Apoptosis of Human Melanocytes in Three-Dimensional Culture
Source: Biomed Res Int. 2021 Jul 20;2021:8463161. doi: 10.1155/2021/8463161 (PMC8315846; doi:10.1155/2021/8463161)
Supplement: Supplementary Materials — Data content comprises assigned gene attribution and protein characterization according to the obtained proteomic analyses and table of semiquantitative distribution of the defined proteins (green color indicates the most abundant; red color indicates the least abundant proteins among analyzed cell cultures; semiquantitative data are estimated in linear and logarithmic scales). Data are deposited in the Mendeley Data Repository and available at the following link: doi:10.17632/nmrfyymrrn.2. [file 8463161.f1.pdf]

SUPPLEMENTARY MATERIAL

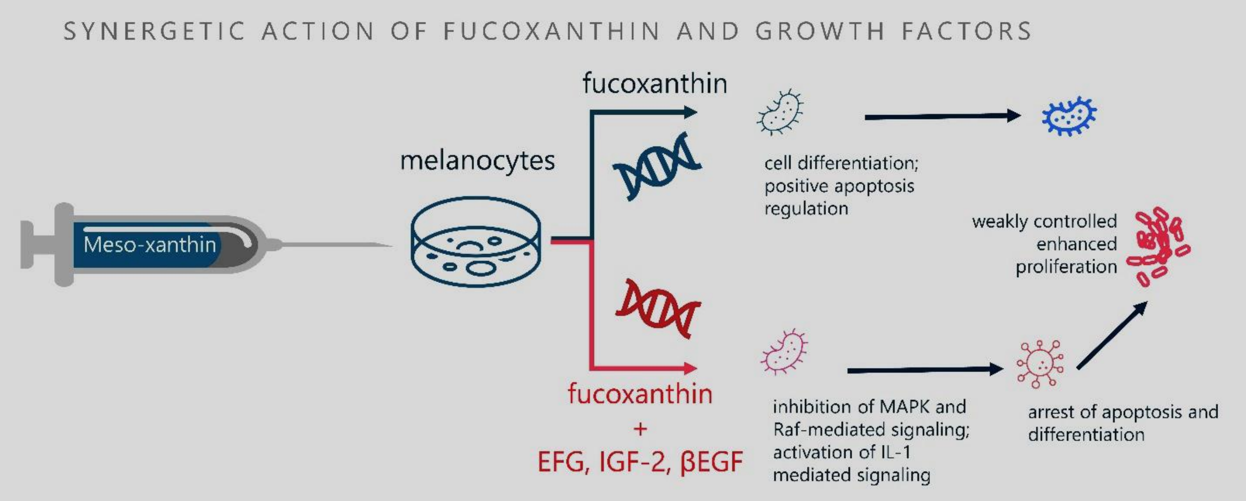

Data are represented as a complete proteome shared between assayed melanocytes cultured in monolayer (M2319) and in spheroids before (Nm01) and after (Am02) exposure to Meso-Xanthin F199™. Data content comprises of assigned genes attribution, proteins characterization according to the obtained proteomic analyzes and table of semi-quantitative distribution of the defined proteins (green color indicates the most abundant; red color indicates the least abundant proteins among analyzed cell cultures; semi-quantitative data are estimated in linear and logarithmic scales). Data availability link (in Mendeley Data Repository): http://dx.doi.org/10.17632/nmrfyymrrn.2

RELATED TO “MAPK AND NOTCH-MEDIATED EFFECTS OF MESO-XANTHIN F199 COMPOUNDS ON PROLIFERATIVE ACTIVITY AND APOPTOSIS OF HUMAN MELANOCYTES IN THREE-DIMENSIONAL CULTURE”.

IRINA N. SABURINA, IRINA M. ZURINA, NASTASIA V. KOSHELEVA, ANASTASIYA A. GORKUN, ELENA N. VOLKOVA, OLGA S. GRINAKOVSKAYA, ANTON S. RYBAKOV, ANNA L. KAYSHEVA, ARTHUR T. KOPYLOV, SERGEY G. MOROZOV

Proetome Diference between 3D melanocytes before (Nm-01) and after (Am-02) exposure to Meso-Xanthin F199

							FOLDS CHANGE, LINEAR scale		
Main Accession	Description	Gene	Ensembl Gene ID	Chromosome	GO Accession	MW [kDa]	NM-01	AM-02	p-value
P31946	14-3-3 protein beta/alpha	1433B	ENSG00000166913	20	GO:0006461, GO:	28.065	0.71	0.50	3.98E-05
P62258	14-3-3 protein epsilon	1433E	ENSG00000108953	17	GO:0040011, GO:	29.155	1.03	1.14	5.67E-04
Q04917	14-3-3 protein eta	1433F	ENSG00000128245	22	GO:0009056, GO:	28.201	2.65	1.53	2.95E-04
P61981	14-3-3 protein gamma	1433G	ENSG00000170027	7	GO:0022607, GO:	28.285	1.49	1.04	2.19E-04
P27348	14-3-3 protein theta	1433T	ENSG00000134308	2	GO:0008150, GO:	27.747	1.25	0.80	3.34E-05
P63104	14-3-3 protein zeta/delta	1433Z	ENSG00000164924	8	GO:0022607, GO:	27.728	1.48	1.19	1.17E-04
P30153	Serine/threonine-protein phosphatase 2A	2AAA	ENSG00000105568	19	GO:0007010, GO:	65.267	0.92	0.70	2.36E-04
Q13541	Eukaryotic translation initiation factor 4E-b	4EBP1	ENSG00000187840	8	GO:0008150, GO:	12.572	2.90	3.59	2.25E-05
P21589	5'-nucleotidase	5NTD	ENSG00000135318	6		63.327	2.50	4.90	2.36E-04
P52209	6-phosphogluconate dehydrogenase, deca	6PGD	ENSG00000142657	1		53.106	0.84	0.63	4.15E-05
P53396	ATP-citrate synthase	ACLY	ENSG00000131473	17		120.762	0.59	0.80	9.15E-05
P62736	Actin, aortic smooth muscle	ACTA	ENSG00000107796	10		41.982	1.84	1.92	6.04E-05
P60709	Actin, cytoplasmic 1	ACTB	ENSG00000075624	7	GO:0007010, GO:	41.710	1.13	1.09	2.18E-04
Q562R1	Beta-actin-like protein 2	ACTBL	ENSG00000169067	5	GO:0008150, GO:	41.976	0.93	1.01	7.81E-05
P63261	Actin, cytoplasmic 2	ACTG	ENSG00000184009	17	GO:0007010, GO:	41.766	0.83	0.87	1.47E-04
P12814	Alpha-actinin-1	ACTN1	ENSG00000072110	14		102.993	1.31	1.42	1.25E-04
O43707	Alpha-actinin-4	ACTN4	ENSG00000282844	CHR_HG26_PAT	GO:0007010, GO:	104.788	1.03	1.23	1.61E-04
P05141	ADP/ATP translocase 2	ADT2	ENSG00000005022	X		32.831	0.31	0.76	3.27E-03
Q09666	Neuroblast differentiation-associated prote	AHNK	ENSG00000124942	11	GO:0008150, GO:	628.699	2.95	5.53	3.55E-04
P04075	Fructose-bisphosphate aldolase A	ALDOA	ENSG00000149925	16		39.395	0.38	0.27	1.71E-04
P09972	Fructose-bisphosphate aldolase C	ALDOC	ENSG00000109107	17	GO:0009056, GO:	39.431	0.72	0.83	5.41E-04
P04083	Annexin A1	ANXA1	ENSG00000135046	9	GO:0016887, GO:	38.690	1.18	1.52	7.49E-05
P07355	Annexin A2	ANXA2	ENSG00000182718	15		38.580	1.49	2.17	9.62E-05
P08758	Annexin A5	ANXA5	ENSG00000164111	4	GO:0008150, GO:	35.914	1.00	1.13	1.14E-03
P08133	Annexin A6	ANXA6	ENSG00000197043	5	GO:0008150, GO:	75.826	1.82	1.66	4.06E-05
P20073	Annexin A7	ANXA7	ENSG00000138279	10		52.706	3.39	1.20	3.78E-04
O95782	AP-2 complex subunit alpha-1	AP2A1	ENSG00000196961	19		107.478	5.14	3.59	6.05E-04
P27695	DNA- lyase	APEX1	ENSG00000100823	14	GO:0040011, GO:	35.532	0.74	0.78	6.24E-05
P07741	Adenine phosphoribosyltransferase	APT	ENSG00000198931	16	GO:0008150, GO:	19.595	2.90	1.20	1.39E-04
O15143	Actin-related protein 2/3 complex subunit	ARC1B	ENSG00000130429	7	GO:0008150, GO:	40.923	1.04	1.26	9.87E-05
P61204	ADP-ribosylation factor 3	ARF3	ENSG00000134287	12		20.588	0.76	0.90	1.37E-04
P18085	ADP-ribosylation factor 4	ARF4	ENSG00000168374	3	GO:0040011, GO:	20.498	1.72	2.03	2.12E-05
P40616	ADP-ribosylation factor-like protein 1	ARL1	ENSG00000120805	12		20.404	0.39	1.20	4.06E-05
P61160	Actin-related protein 2	ARP2	ENSG00000138071	2		44.732	1.74	1.02	4.67E-05
P61158	Actin-related protein 3	ARP3	ENSG00000115091	2	GO:0007010, GO:	47.341	1.05	1.50	2.07E-05
O15144	Actin-related protein 2/3 complex subunit	ARPC2	ENSG00000163466	2	GO:0007010, GO:	34.311	4.27	1.20	1.25E-04
O15145	Actin-related protein 2/3 complex subunit	ARPC3	ENSG00000111229	12	GO:0008150, GO:	20.533	2.70	1.03	1.34E-04

P59998	Actin-related protein 2/3 complex subunit 4	ARPC4	ENSG00000241553	3		19.654	0.79	0.52	5.45E-05
Q12797	Aspartyl/asparaginyl beta-hydroxylase	ASPH	ENSG00000198363	8		85.809	0.43	1.00	0.00E+00
P05023	Sodium/potassium-transporting ATPase su	AT1A1	ENSG00000163399	1		112.824	0.50	1.36	1.79E-04
P25705	ATP synthase subunit alpha, mitochondria	ATPA				59.714	0.54	0.34	2.08E-04
P06576	ATP synthase subunit beta, mitochondrial	ATPB			GO:0016887, GO:	56.525	0.64	0.54	8.93E-05
P51572	B-cell receptor-associated protein 31	BAP31	ENSG00000185825	X		27.974	0.50	0.54	1.41E-05
P80723	Brain acid soluble protein 1	BASP1	ENSG00000176788	5		22.680	3.46	5.35	6.45E-05
P11021	Endoplasmic reticulum chaperone BiP	BIP	ENSG00000044574	9	GO:0016887, GO:	72.288	0.99	1.95	2.22E-04
Q07021	Complement component 1 Q subcompone	C1QBP	ENSG00000108561	17	GO:0040011, GO:	31.343	0.19	0.48	1.25E-04
P0DP23	Calmodulin-1	CALM1	ENSG00000198668	14		16.827	0.95	1.50	2.71E-05
P27797	Calreticulin	CALR	ENSG00000179218	19	GO:0007010, GO:	48.112	0.68	0.72	1.10E-04
O43852	Calumenin	CALU	ENSG00000128595	7		37.084	1.32	2.25	1.57E-04
P27824	Calnexin	CALX	ENSG00000283777	CHR_HG30_PATCH		67.526	0.95	1.37	5.01E-05
P17655	Calpain-2 catalytic subunit	CAN2	ENSG00000162909	1		79.945	1.34	0.94	2.06E-04
Q01518	Adenylyl cyclase-associated protein 1	CAP1	ENSG00000131236	1	GO:0007010, GO:	51.869	1.26	1.45	2.39E-04
P40121	Macrophage-capping protein	CAPG	ENSG00000042493	2		38.474	2.32	1.79	4.35E-05
P47756	F-actin-capping protein subunit beta	CAPZB	ENSG00000077549	1		31.331	1.26	1.09	1.88E-04
P07858	Cathepsin B	CATB	ENSG00000164733	8	GO:0009056, GO:	37.797	0.96	0.57	1.49E-05
P07339	Cathepsin D	CATD	ENSG00000117984	11	GO:0008150, GO:	44.524	1.03	0.82	8.15E-05
Q03135	Caveolin-1	CAV1	ENSG00000105974	7	GO:0022607, GO:	20.458	5.32	6.18	1.71E-04
Q6NZI2	Caveolae-associated protein 1	CAVN1	ENSG00000177469	17	GO:0008150, GO:	43.450	1.82	3.56	3.52E-05
P52907	F-actin-capping protein subunit alpha-1	CAZA1	ENSG00000116489	1	GO:0008150, GO:	32.902	1.23	0.73	3.01E-05
P13987	CD59 glycoprotein	CD59	ENSG00000085063	11		14.168	2.26	2.19	5.34E-05
Q16543	Hsp90 co-chaperone Cdc37	CDC37	ENSG00000105401	19	GO:0009056, GO:	44.440	1.62	1.28	1.71E-04
P60953	Cell division control protein 42 homolog	CDC42	ENSG00000070831	1	GO:0007010, GO:	21.245	2.14	5.78	7.20E-05
P10809	60 kDa heat shock protein, mitochondrial	CH60	ENSG00000144381	2	GO:0008283, GO:	61.016	1.47	2.08	2.05E-05
O75390	Citrate synthase, mitochondrial	CISY	ENSG00000062485	12	GO:0008150, GO:	51.680	0.57	0.14	6.85E-05
Q07065	Cytoskeleton-associated protein 4	CKAP4	ENSG00000136026	12	GO:0008150, GO:	65.983	0.95	0.66	3.26E-04
Q00610	Clathrin heavy chain 1	CLH1	ENSG00000141367	17		191.493	0.98	0.99	1.64E-04
O00299	Chloride intracellular channel protein 1	CLIC1	ENSG00000226248	CHR_HSCHR6_M	GO:0008150, GO:	26.906	0.85	0.97	2.04E-04
Q9Y696	Chloride intracellular channel protein 4	CLIC4	ENSG00000169504	1	GO:0007010, GO:	28.754	1.57	1.00	5.50E-05
Q15417	Calponin-3	CNN3	ENSG00000117519	1		36.391	0.50	1.00	3.26E-04
P08123	Collagen alpha-2 chain	CO1A2	ENSG00000164692	7	GO:0009056, GO:	129.235	3.40	1.15	2.22E-05
P23528	Cofilin-1	COF1	ENSG00000172757	11	GO:0009790, GO:	18.491	1.32	1.39	6.30E-04
P53621	Coatomer subunit alpha	COPA	ENSG00000122218	1		138.258	0.97	3.59	1.57E-04
P53618	Coatomer subunit beta	COPB	ENSG00000129083	11	GO:0008150, GO:	107.074	1.13	0.40	2.13E-05
P35606	Coatomer subunit beta'	COPB2	ENSG00000184432	3		102.422	1.03	0.87	2.22E-05
P48444	Coatomer subunit delta	COPD	ENSG00000095139	11	GO:0008150, GO:	57.174	1.19	1.43	3.78E-04
Q9Y678	Coatomer subunit gamma-1	COPG1	ENSG00000181789	3	GO:0008150, GO:	97.655	0.53	0.82	1.75E-04
Q9ULV4	Coronin-1C	COR1C	ENSG00000110880	12		53.215	1.81	0.75	1.71E-04
Q14019	Coactosin-like protein	COTL1	ENSG00000103187	16	GO:0008150, GO:	15.935	0.88	0.82	2.44E-04
P04632	Calpain small subunit 1	CPNS1	ENSG00000126247	19	GO:0008283, GO:	28.298	1.31	1.84	2.14E-04
P21291	Cysteine and glycine-rich protein 1	CSRP1	ENSG00000159176	1	GO:0008150, GO:	20.554	1.01	0.55	3.17E-05
Q9UJU6	Drebrin-like protein	DBNL	ENSG00000136279	7		48.178	0.82	1.10	9.87E-05

Q14203	Dynactin subunit 1	DCTN1	ENSG00000204843	2		141.607	0.64	0.20	8.15E-05
Q92499	ATP-dependent RNA helicase DDX1	DDX1	ENSG00000079785	2		82.380	5.08	0.60	1.78E-04
O00571	ATP-dependent RNA helicase DDX3X	DDX3X	ENSG00000215301	X		73.198	0.62	1.19	2.01E-04
P60981	Destrin	DEST	ENSG00000125868	20	GO:0005623, GO:	18.493	1.90	2.74	1.04E-03
Q08211	ATP-dependent RNA helicase A	DHX9	ENSG00000135829	1	GO:0016887, GO:	140.869	1.06	0.98	4.15E-05
Q8TF09	Dynein light chain roadblock-type 2	DLRB2	ENSG00000168589	16		10.848	0.73	1.79	1.62E-03
Q16555	Dihydropyrimidinase-related protein 2	DPYL2	ENSG00000092964	8		62.255	1.65	0.71	9.87E-05
Q16643	Drebrin	DREB	ENSG00000113758	5		71.385	3.36	2.19	2.25E-05
O00148	ATP-dependent RNA helicase DDX39A	DX39A	ENSG00000123136	19		49.098	0.79	0.29	6.30E-04
Q14204	Cytoplasmic dynein 1 heavy chain 1	DYHC1	ENSG00000197102	14	GO:0016887, GO:	532.072	2.27	2.59	8.52E-05
P68104	Elongation factor 1-alpha 1	EF1A1	ENSG00000156508	6		50.109	0.96	2.16	1.25E-04
P24534	Elongation factor 1-beta	EF1B	ENSG00000283391	CHR_HSCHR2_	GO:0008150, GO:	24.748	0.77	1.71	5.67E-05
P29692	Elongation factor 1-delta	EF1D	ENSG00000273594	CHR_HSCHR8_3_CTG7		31.103	1.28	1.09	1.39E-04
P26641	Elongation factor 1-gamma	EF1G	ENSG00000254772	11	GO:0008150, GO:	50.087	1.07	1.13	6.31E-05
P13639	Elongation factor 2	EF2	ENSG00000167658	19	GO:0008283, GO:	95.277	1.16	1.31	2.07E-05
Q9NZN4	EH domain-containing protein 2	EHD2	ENSG00000024422	19	GO:0061024, GO:	61.123	0.68	1.44	3.11E-05
Q14152	Eukaryotic translation initiation factor 3 sul	EIF3A	ENSG00000107581	10		166.468	1.21	1.20	5.34E-05
P55884	Eukaryotic translation initiation factor 3 sul	EIF3B	ENSG00000106263	7		92.424	1.77	1.79	3.86E-04
P60228	Eukaryotic translation initiation factor 3 sul	EIF3E	ENSG00000104408	8	GO:0008150, GO:	52.187	1.76	1.52	8.47E-04
Q15370	Elongin-B	ELOB	ENSG00000103363	16		13.125	0.50	0.55	9.38E-05
P50402	Emerin	EMD	ENSG00000102119	X	GO:0008283, GO:	28.976	3.39	2.39	6.93E-05
P06733	Alpha-enolase	ENOA	ENSG00000074800	1	GO:0009056, GO:	47.139	0.73	0.90	6.24E-05
P14625	Endoplasmin	ENPL	ENSG00000166598	12	GO:0007010, GO:	92.411	1.01	0.83	3.78E-04
Q969X5	Endoplasmic reticulum-Golgi intermediate	ERGI1	ENSG00000113719	5		32.571	1.13	0.66	1.22E-04
P30040	Endoplasmic reticulum resident protein 29	ERP29	ENSG00000089248	12	GO:0008150, GO:	28.975	0.77	0.78	4.15E-05
Q01844	RNA-binding protein EWS	EWS	ENSG00000182944	22		68.436	0.97	0.60	1.71E-04
P49327	Fatty acid synthase	FAS	ENSG00000169710	17	GO:0016746, GO:	273.254	1.39	1.49	3.81E-05
P02765	Alpha-2-HS-glycoprotein	FETUA	ENSG00000145192	3	GO:0008150, GO:	39.316	1.61	0.64	1.65E-04
Q9Y3D6	Mitochondrial fission 1 protein	FIS1	ENSG00000214253	7	GO:0006461, GO:	16.927	0.77	1.20	4.01E-04
Q96AY3	Peptidyl-prolyl cis-trans isomerase FKBP1	FKB10	ENSG00000141756	17	GO:0008150, GO:	64.204	0.72	1.56	6.94E-04
P62942	Peptidyl-prolyl cis-trans isomerase FKBP1	FKB1A	ENSG00000088832	20	GO:0006461, GO:	11.943	0.24	0.90	2.81E-04
P26885	Peptidyl-prolyl cis-trans isomerase FKBP2	FKBP2	ENSG00000173486	11	GO:0005623, GO:	15.639	1.45	1.20	9.07E-05
O95302	Peptidyl-prolyl cis-trans isomerase FKBP9	FKBP9	ENSG00000122642	7		63.044	1.03	0.43	4.79E-05
P21333	Filamin-A	FLNA	ENSG00000196924	X		280.564	1.01	1.54	4.51E-04
O75369	Filamin-B	FLNB	ENSG00000136068	3		277.990	1.61	1.17	6.24E-05
Q14315	Filamin-C	FLNC	ENSG00000128591	7		290.841	0.91	1.34	3.77E-05
Q16658	Fascin	FSCN1	ENSG00000075618	7	GO:0008283, GO:	54.496	1.03	0.88	1.04E-04
Q13283	Ras GTPase-activating protein-binding pro	G3BP1	ENSG00000145907	5	GO:0016887, GO:	52.132	0.92	1.00	1.71E-04
P04406	Glyceraldehyde-3-phosphate dehydrogena	G3P	ENSG00000111640	12	GO:0007010, GO:	36.030	1.00	1.04	6.93E-05
P11413	Glucose-6-phosphate 1-dehydrogenase	G6PD	ENSG00000160211	X		59.219	3.39	1.37	4.73E-04
P06744	Glucose-6-phosphate isomerase	G6PI	ENSG00000282019	CHR_HSCHR19_2_CTG3_1		63.107	0.68	0.81	4.30E-04
Q14697	Neutral alpha-glucosidase AB	GANAB	ENSG00000089597	11		106.807	1.33	0.86	4.79E-05
P41250	Glycine--tRNA ligase	GARS	ENSG00000106105	7	GO:0008150, GO:	83.113	2.18	2.63	4.79E-05
P62873	Guanine nucleotide-binding protein G/G/G	GBB1	ENSG00000078369	1		37.353	1.30	0.80	1.10E-04

Q9UBI6	Guanine nucleotide-binding protein G/G/G	GBG12	ENSG00000172380	1	GO:0008150, GO:	8.001	1.29	2.73	2.00E-04
P50395	Rab GDP dissociation inhibitor beta	GDIB	ENSG00000057608	10	GO:0061024, GO:	50.631	1.19	1.29	4.45E-05
P52565	Rho GDP-dissociation inhibitor 1	GDIR1	ENSG00000141522	17	GO:0008150, GO:	23.193	1.27	0.66	4.92E-05
P06396	Gelsolin	GELS	ENSG00000148180	9		85.644	0.74	0.51	1.34E-04
Q04446	1,4-alpha-glucan-branching enzyme	GLGB	ENSG00000114480	3	GO:0008150, GO:	80.423	0.37	0.55	8.24E-05
P35754	Glutaredoxin-1	GLRX1	ENSG00000173221	5	GO:0008150, GO:	11.768	0.28	0.17	6.45E-05
P14314	Glucosidase 2 subunit beta	GLU2B	ENSG00000130175	19		59.388	0.77	0.84	4.62E-05
P04899	Guanine nucleotide-binding protein G subu	GNAI2	ENSG00000114353	3		40.425	5.32	4.78	4.79E-05
P38646	Stress-70 protein, mitochondrial	GRP75	ENSG00000113013	5	GO:0008150, GO:	73.635	0.92	0.79	4.25E-05
P78417	Glutathione S-transferase omega-1	GSTO1	ENSG00000148834	10		27.548	1.25	1.20	7.49E-05
P09211	Glutathione S-transferase P	GSTP1	ENSG00000084207	11	GO:0008283, GO:	23.341	0.68	0.69	6.19E-04
Q8NBJ5	Procollagen galactosyltransferase 1	GT251	ENSG00000130309	19	GO:0005623, GO:	71.590	1.36	1.43	1.57E-04
P16401	Histone H1.5	H15	ENSG00000184357	6	GO:0008150, GO:	22.566	0.85	0.68	2.72E-04
P04908	Histone H2A type 1-B/E	H2A1B	ENSG00000278463	6		14.127	1.23	0.10	2.70E-04
O60814	Histone H2B type 1-K	H2B1K	ENSG00000197903	6		13.882	0.79	0.73	4.67E-05
P68431	Histone H3.1	H31	ENSG00000275714	6		15.394	0.17	0.21	1.13E-04
P62805	Histone H4	H4	ENSG00000278637	6	GO:0006461, GO:	11.360	0.98	1.18	1.02E-03
Q9NRV9	Heme-binding protein 1	HEBP1	ENSG00000013583	12	GO:0008150, GO:	21.084	0.41	0.35	2.63E-05
P09429	High mobility group protein B1	HMGB1	ENSG00000189403	13		24.878	1.11	0.69	1.69E-03
O14979	Heterogeneous nuclear ribonucleoprotein	HNRDL	ENSG00000152795	4		46.409	0.78	0.58	2.87E-05
P31943	Heterogeneous nuclear ribonucleoprotein	HNRH1	ENSG00000169045	5	GO:0008150, GO:	49.198	0.68	2.21	3.28E-05
P55795	Heterogeneous nuclear ribonucleoprotein	HNRH2	ENSG00000126945	X	GO:0008150, GO:	49.232	0.37	0.31	3.29E-04
P07910	Heterogeneous nuclear ribonucleoproteins	HNRPC	ENSG00000092199	14		33.650	0.98	0.62	8.52E-04
P61978	Heterogeneous nuclear ribonucleoprotein	HNRPK	ENSG00000165119	9		50.944	0.64	0.41	3.15E-04
P14866	Heterogeneous nuclear ribonucleoprotein	HNRPL	ENSG00000282947	CHR_HG26_PAT	GO:0008150, GO:	64.092	0.84	0.43	4.92E-05
P52272	Heterogeneous nuclear ribonucleoprotein	HNRPM	ENSG00000099783	19		77.464	2.62	1.45	1.45E-04
O60506	Heterogeneous nuclear ribonucleoprotein	HNRPQ	ENSG00000135316	6		69.560	1.13	0.91	7.70E-04
Q00839	Heterogeneous nuclear ribonucleoprotein	HNRPU	ENSG00000153187	1		90.528	0.91	0.50	5.16E-04
P0DMV8	Heat shock 70 kDa protein 1A	HS71A	ENSG00000235941	CHR_HSCHR6_MHC_APD_CTG1		70.009	0.94	0.87	4.35E-05
P07900	Heat shock protein HSP 90-alpha	HS90A	ENSG00000080824	14		84.607	0.52	0.60	8.83E-04
P08238	Heat shock protein HSP 90-beta	HS90B	ENSG00000096384	6	GO:0009056, GO:	83.212	0.57	0.68	3.15E-04
P34932	Heat shock 70 kDa protein 4	HSP74	ENSG00000170606	5	GO:0008150, GO:	94.271	1.25	0.90	2.68E-05
P11142	Heat shock cognate 71 kDa protein	HSP7C	ENSG00000109971	11	GO:0016887, GO:	70.854	0.73	0.98	3.89E-05
P04792	Heat shock protein beta-1	HSPB1	ENSG00000106211	7	GO:0009056, GO:	22.768	1.48	2.34	6.57E-04
P05198	Eukaryotic translation initiation factor 2 sul	IF2A	ENSG00000134001	14	GO:0008150, GO:	36.089	0.48	0.30	6.33E-03
P60842	Eukaryotic initiation factor 4A-I	IF4A1	ENSG00000161960	17	GO:0016887, GO:	46.125	0.60	0.69	3.04E-04
Q04637	Eukaryotic translation initiation factor 4 ga	IF4G1	ENSG00000114867	3		175.382	5.20	8.31	5.64E-07
Q15056	Eukaryotic translation initiation factor 4H	IF4H	ENSG00000106682	7		27.368	1.06	1.04	2.29E-05
P63241	Eukaryotic translation initiation factor 5A-1	IF5A1	ENSG00000132507	17		16.821	1.02	1.57	1.33E-04
Q12906	Interleukin enhancer-binding factor 3	ILF3	ENSG00000129351	19		95.279	0.38	0.78	1.06E-04
Q14974	Importin subunit beta-1	IMB1	ENSG00000108424	17	GO:0007010, GO:	97.108	1.50	1.52	0.00E+00
O00410	Importin-5	IPO5	ENSG00000065150	13		123.550	1.42	0.95	2.70E-04
Q15181	Inorganic pyrophosphatase	IPYR	ENSG00000180817	10	GO:0008150, GO:	32.639	1.76	1.25	1.10E-04
P46940	Ras GTPase-activating-like protein IQGAP	IQGA1	ENSG00000140575	15	GO:0040011, GO:	189.134	2.33	2.68	3.34E-04

P05556	Integrin beta-1	ITB1	ENSG00000150093	10	GO:0009790, GO:	88.357	1.93	1.79	5.52E-04
P00568	Adenylate kinase isoenzyme 1	KAD1	ENSG00000106992	9	GO:0008150, GO:	21.621	2.26	1.33	1.78E-04
P54819	Adenylate kinase 2, mitochondrial	KAD2	ENSG00000004455	1		26.461	0.74	1.08	2.14E-05
P30085	UMP-CMP kinase	KCY	ENSG00000162368	1		22.208	0.85	0.97	1.71E-04
Q07866	Kinesin light chain 1	KLC1	ENSG00000126214	14		65.269	1.16	2.69	3.17E-05
P14618	Pyruvate kinase PKM	KPYM	ENSG00000067225	15		57.900	0.83	0.42	9.51E-06
P11279	Lysosome-associated membrane glycopro	LAMP1	ENSG00000185896	13		44.854	1.98	0.98	1.19E-04
P13473	Lysosome-associated membrane glycopro	LAMP2	ENSG00000005893	X		44.932	1.29	0.89	4.30E-05
P00338	L-lactate dehydrogenase A chain	LDHA	ENSG00000134333	11		36.665	0.88	0.41	1.21E-04
P07195	L-lactate dehydrogenase B chain	LDHB	ENSG00000111716	12	GO:0008150, GO:	36.615	0.85	0.40	8.15E-05
P09382	Galectin-1	LEG1	ENSG00000100097	22	GO:0006464, GO:	14.706	1.53	1.10	6.63E-04
Q04760	Lactoylglutathione lyase	LGUL	ENSG00000124767	6		20.764	1.42	0.28	2.81E-04
P02545	Prelamin-A/C	LMNA	ENSG00000160789	1		74.095	1.75	0.92	2.16E-04
Q96AG4	Leucine-rich repeat-containing protein 59	LRC59	ENSG00000108829	17	GO:0005635, GO:	34.909	1.07	1.70	2.97E-05
O43504	Ragulator complex protein LAMTOR5	LTOR5	ENSG00000134248	1	GO:0008150, GO:	9.608	0.64	1.59	9.54E-05
P46821	Microtubule-associated protein 1B	MAP1B	ENSG00000131711	5	GO:0008150, GO:	270.468	5.63	4.32	8.93E-05
P27816	Microtubule-associated protein 4	MAP4	ENSG00000047849	3		120.930	1.20	0.65	6.01E-04
P29966	Myristoylated alanine-rich C-kinase substr	MARCS	ENSG00000277443	6	GO:0008150, GO:	31.536	0.79	0.54	1.19E-05
P43243	Matrin-3	MATR3	ENSG00000015479	5	GO:0005635, GO:	94.565	0.53	0.33	4.15E-05
P40925	Malate dehydrogenase, cytoplasmic	MDHC	ENSG00000014641	2		36.403	0.88	0.69	1.04E-04
P40926	Malate dehydrogenase, mitochondrial	MDHM	ENSG00000146701	7	GO:0008150, GO:	35.481	0.58	0.58	3.81E-05
P14174	Macrophage migration inhibitory factor	MIF	ENSG00000240972	22	GO:0008283, GO:	12.468	0.54	0.22	5.87E-04
P19105	Myosin regulatory light chain 12A	ML12A	ENSG00000101608	18	GO:0008150, GO:	19.781	1.53	1.57	5.30E-04
O14950	Myosin regulatory light chain 12B	ML12B	ENSG00000118680	18	GO:0008150, GO:	19.767	1.32	1.37	1.34E-04
P26038	Moesin	MOES	ENSG00000147065	X	GO:0008283, GO:	67.778	0.74	0.90	1.50E-04
Q00325	Phosphate carrier protein, mitochondrial	MPCP	ENSG00000075415	12		40.069	0.79	0.30	8.93E-05
Q14764	Major vault protein	MVP	ENSG00000013364	16	GO:0008150, GO:	99.266	0.72	0.56	1.39E-04
Q969H8	Myeloid-derived growth factor	MYDGF	ENSG00000074842	19	GO:0008283, GO:	18.783	0.69	1.71	9.93E-04
P35580	Myosin-10	MYH10	ENSG00000133026	17		228.858	1.76	0.93	2.83E-05
Q7Z406	Myosin-14	MYH14	ENSG00000105357	19		227.732	1.51	0.92	2.05E-04
P35579	Myosin-9	MYH9	ENSG00000100345	22	GO:0016887, GO:	226.392	1.29	0.92	1.28E-04
P60660	Myosin light polypeptide 6	MYL6	ENSG00000092841	12		16.919	1.49	1.29	2.17E-04
O00159	Unconventional myosin-Ic	MYO1C	ENSG00000197879	17		121.606	2.18	1.49	5.67E-04
E9PAV3	Nascent polypeptide-associated complex s	NACAM	ENSG00000196531	12		205.295	1.93	1.79	2.60E-04
P43490	Nicotinamide phosphoribosyltransferase	NAMPT	ENSG00000105835	7	GO:0008283, GO:	55.487	0.62	0.11	1.57E-04
P00387	NADH-cytochrome b5 reductase 3	NB5R3	ENSG00000100243	22		34.213	3.25	3.92	9.38E-05
P15531	Nucleoside diphosphate kinase A	NDKA	ENSG00000239672	17		17.138	3.08	1.32	1.66E-04
P22392	Nucleoside diphosphate kinase B	NDKB	ENSG00000243678	17		17.287	2.76	2.40	4.06E-04
Q92597	Protein NDRG1	NDRG1	ENSG00000104419	8	GO:0008283, GO:	42.808	3.87	3.59	1.62E-03
P40261	Nicotinamide N-methyltransferase	NNMT	ENSG00000166741	11	GO:0008150, GO:	29.555	0.55	0.64	1.49E-04
Q15233	Non-POU domain-containing octamer-bind	NONO	ENSG00000147140	X	GO:0008150, GO:	54.197	0.54	0.56	2.70E-04
P55209	Nucleosome assembly protein 1-like 1	NP1L1	ENSG00000187109	12		45.346	1.81	1.83	1.77E-04
P06748	Nucleophosmin	NPM	ENSG00000181163	5		32.555	1.50	1.69	2.98E-04
P61970	Nuclear transport factor 2	NTF2	ENSG00000102898	16	GO:0008150, GO:	14.469	0.31	0.33	5.42E-05

P19338	Nucleolin	NUCL	ENSG00000115053	2	GO:0008150, GO:	76.568	0.51	0.55	6.86E-04
P04181	Ornithine aminotransferase, mitochondrial	OAT	ENSG00000065154	10	GO:0008150, GO:	48.504	0.52	0.59	2.03E-05
P39656	Dolichyl-diphosphooligosaccharide--protei	OST48	ENSG00000244038	1		50.769	1.38	0.51	3.46E-05
Q32P28	Prolyl 3-hydroxylase 1	P3H1	ENSG00000117385	1		83.341	0.40	0.90	6.69E-05
P13674	Prolyl 4-hydroxylase subunit alpha-1	P4HA1	ENSG00000122884	10		61.011	0.61	0.29	1.17E-04
Q9UQ80	Proliferation-associated protein 2G4	PA2G4	ENSG00000170515	12		43.759	0.97	1.49	1.04E-04
P05120	Plasminogen activator inhibitor 2	PAI2	ENSG00000197632	18	GO:0008150, GO:	46.566	0.71	0.94	2.36E-04
Q99497	Protein/nucleic acid deglycase DJ-1	PARK7	ENSG00000116288	1	GO:0006461, GO:	19.878	0.64	0.95	2.60E-04
P49023	Paxillin	PAXI	ENSG00000089159	12		64.464	2.26	0.80	4.25E-05
Q15365	Poly-binding protein 1	PCBP1	ENSG00000169564	2		37.474	5.37	0.80	7.42E-05
P07237	Protein disulfide-isomerase	PDIA1	ENSG00000185624	17	GO:0022607, GO:	57.081	1.87	1.88	9.38E-05
P30101	Protein disulfide-isomerase A3	PDIA3	ENSG00000167004	15	GO:0005576, GO:	56.747	0.60	0.70	1.88E-04
P13667	Protein disulfide-isomerase A4	PDIA4	ENSG00000155660	7	GO:0008150, GO:	72.887	0.29	0.44	3.66E-05
Q15084	Protein disulfide-isomerase A6	PDIA6	ENSG00000143870	2		48.091	0.88	1.05	2.33E-05
Q15121	Astrocytic phosphoprotein PEA-15	PEA15	ENSG00000162734	1		15.031	0.29	0.32	1.14E-04
P30086	Phosphatidylethanolamine-binding protein	PEBP1	ENSG00000089220	12	GO:0008150, GO:	21.044	0.74	0.54	1.50E-05
Q01813	ATP-dependent 6-phosphofructokinase, p	PFKAP	ENSG00000067057	10		85.542	1.13	0.60	4.40E-05
P18669	Phosphoglycerate mutase 1	PGAM1	ENSG00000171314	10	GO:0019748, GO:	28.786	1.61	1.74	2.80E-04
P00558	Phosphoglycerate kinase 1	PGK1	ENSG00000102144	X		44.586	0.87	0.90	1.57E-04
P36871	Phosphoglucomutase-1	PGM1	ENSG00000079739	1	GO:0008150, GO:	61.411	1.59	0.81	7.81E-05
O00264	Membrane-associated progesterone recep	PGRC1	ENSG00000101856	X	GO:0008150, GO:	21.658	0.83	1.10	2.48E-04
P35232	Prohibitin	PHB	ENSG00000167085	17		29.786	0.60	0.30	3.17E-05
Q99623	Prohibitin-2	PHB2	ENSG00000215021	12		33.276	0.42	0.63	7.20E-05
Q9NRX4	14 kDa phosphohistidine phosphatase	PHP14	ENSG00000054148	9		13.824	0.97	0.45	1.25E-04
Q15149	Plectin	PLEC	ENSG00000178209	8		531.466	0.20	0.40	2.05E-04
O60664	Perilipin-3	PLIN3	ENSG00000105355	19		47.046	0.68	0.46	2.70E-04
O00469	Procollagen-lysine,2-oxoglutarate 5-dioxyg	PLOD2	ENSG00000152952	3	GO:0008150, GO:	84.632	0.60	0.33	1.57E-04
P13797	Plastin-3	PLST	ENSG00000102024	X		70.766	0.94	1.18	6.69E-05
P62937	Peptidyl-prolyl cis-trans isomerase A	PPIA	ENSG00000196262	7	GO:0040011, GO:	18.001	0.62	0.61	2.36E-04
P23284	Peptidyl-prolyl cis-trans isomerase B	PPIB	ENSG00000166794	15	GO:0008150, GO:	23.728	0.55	0.54	3.15E-04
O75915	PRA1 family protein 3	PRAF3	ENSG00000144746	3	GO:0008150, GO:	21.600	1.45	1.20	1.21E-04
Q06830	Peroxiredoxin-1	PRDX1	ENSG00000117450	1	GO:0008283, GO:	22.096	1.17	0.71	1.50E-04
P32119	Peroxiredoxin-2	PRDX2	ENSG00000167815	19	GO:0008150, GO:	21.878	0.89	0.53	2.52E-04
P30041	Peroxiredoxin-6	PRDX6	ENSG00000117592	1	GO:0008150, GO:	25.019	0.80	0.82	2.09E-04
P78527	DNA-dependent protein kinase catalytic su	PRKDC	ENSG00000253729	8		468.788	2.67	1.06	7.53E-04
P07737	Profilin-1	PROF1	ENSG00000108518	17	GO:0009790, GO:	15.045	0.62	0.95	5.19E-05
P17980	26S proteasome regulatory subunit 6A	PRS6A	ENSG00000165916	11	GO:0016887, GO:	49.172	0.48	2.39	1.38E-04
P43686	26S proteasome regulatory subunit 6B	PRS6B	ENSG00000281221	CHR_HG2021_PATCH		47.337	1.45	2.99	5.50E-05
P35998	26S proteasome regulatory subunit 7	PRS7	ENSG00000161057	7		48.603	1.17	1.54	3.22E-05
P62195	26S proteasome regulatory subunit 8	PRS8	ENSG00000087191	17		45.597	0.48	0.30	2.12E-05
P55786	Puromycin-sensitive aminopeptidase	PSA	ENSG00000141279	17		103.211	1.07	0.80	0.00E+00
O14818	Proteasome subunit alpha type-7	PSA7	ENSG00000101182	20		27.870	0.35	0.21	3.74E-05
P20618	Proteasome subunit beta type-1	PSB1	ENSG00000281184	CHR_HSCHR6_1	GO:0009056, GO:	26.472	0.57	0.42	3.28E-05
Q9UNM6	26S proteasome non-ATPase regulatory s	PSD13	ENSG00000185627	11		42.918	2.13	1.91	1.57E-05

Q13200	26S proteasome non-ATPase regulatory s	PSMD2	ENSG00000175166	3	GO:0009056, GO:	100.136	2.19	1.37	2.46E-04
Q06323	Proteasome activator complex subunit 1	PSME1	ENSG00000092010	14		28.705	0.38	0.12	9.38E-05
Q9UL46	Proteasome activator complex subunit 2	PSME2	ENSG00000100911	14	GO:0009056, GO:	27.384	0.74	0.83	2.70E-04
P26599	Polypyrimidine tract-binding protein 1	PTBP1	ENSG00000011304	19		57.186	0.64	0.70	2.63E-05
Q16647	Prostacyclin synthase	PTGIS	ENSG00000124212	20	GO:0048856, GO:	57.068	0.66	0.36	2.36E-04
P06454	Prothymosin alpha	PTMA	ENSG00000187514	2		12.196	1.00	0.74	9.38E-05
P20962	Parathymosin	PTMS	ENSG00000159335	12	GO:0005634, GO:	11.523	0.55	0.34	1.14E-04
P61026	Ras-related protein Rab-10	RAB10	ENSG00000084733	2	GO:0008283, GO:	22.527	1.01	2.30	5.97E-03
P62820	Ras-related protein Rab-1A	RAB1A	ENSG00000138069	2		22.663	0.60	0.44	1.75E-03
P61019	Ras-related protein Rab-2A	RAB2A	ENSG00000104388	8		23.531	1.19	1.88	9.87E-05
P51148	Ras-related protein Rab-5C	RAB5C	ENSG00000108774	17		23.468	1.34	0.41	6.30E-06
P51149	Ras-related protein Rab-7a	RAB7A	ENSG00000075785	3	GO:0009056, GO:	23.475	0.85	0.90	6.17E-05
P63244	Receptor of activated protein C kinase 1	RACK1	ENSG00000204628	5	GO:0009790, GO:	35.055	2.66	1.44	1.10E-04
P62826	GTP-binding nuclear protein Ran	RAN	ENSG00000132341	12	GO:0007010, GO:	24.408	1.57	2.24	5.67E-04
P61224	Ras-related protein Rap-1b	RAP1B	ENSG00000127314	12		20.812	7.98	1.20	2.36E-04
P62491	Ras-related protein Rab-11A	RB11A	ENSG00000103769	15	GO:0007010, GO:	24.378	0.73	0.90	3.78E-04
Q15293	Reticulocalbin-1	RCN1	ENSG00000049449	11	GO:0009790, GO:	38.866	0.42	0.64	5.50E-05
P54727	UV excision repair protein RAD23 homolo	RD23B	ENSG00000119318	9		43.145	0.73	0.90	1.01E-03
Q07960	Rho GTPase-activating protein 1	RHG01	ENSG00000175220	11	GO:0008150, GO:	50.404	1.13	3.39	0.00E+00
P13489	Ribonuclease inhibitor	RINI	ENSG00000276230	CHR_HSCHR11_	GO:0008150, GO:	49.941	0.67	0.55	2.79E-05
P27635	60S ribosomal protein L10	RL10	ENSG00000147403	X	GO:0009056, GO:	24.588	3.91	3.54	6.24E-05
P62906	60S ribosomal protein L10a	RL10A	ENSG00000198755	6	GO:0008150, GO:	24.816	1.22	2.45	4.06E-05
P62913	60S ribosomal protein L11	RL11	ENSG00000142676	1	GO:0009056, GO:	20.240	0.52	1.16	2.22E-04
P30050	60S ribosomal protein L12	RL12	ENSG00000197958	9	GO:0009056, GO:	17.808	0.71	1.75	1.71E-04
P26373	60S ribosomal protein L13	RL13	ENSG00000167526	16	GO:0008150, GO:	24.247	1.16	1.00	1.98E-04
P50914	60S ribosomal protein L14	RL14	ENSG00000188846	3	GO:0008150, GO:	23.417	1.45	0.30	3.69E-04
P61313	60S ribosomal protein L15	RL15	ENSG00000174748	3	GO:0008150, GO:	24.131	1.97	1.75	5.85E-05
P18621	60S ribosomal protein L17	RL17	ENSG00000265681	18		21.383	0.64	0.80	1.71E-04
Q07020	60S ribosomal protein L18	RL18	ENSG00000063177	19		21.621	2.20	1.48	1.45E-04
Q02543	60S ribosomal protein L18a	RL18A	ENSG00000105640	19	GO:0008150, GO:	20.749	1.06	0.48	1.45E-04
P84098	60S ribosomal protein L19	RL19	ENSG00000108298	17	GO:0008150, GO:	23.451	0.32	0.95	1.94E-05
P46778	60S ribosomal protein L21	RL21	ENSG00000122026	13	GO:0008150, GO:	18.553	1.13	3.06	6.86E-04
P35268	60S ribosomal protein L22	RL22	ENSG00000116251	1	GO:0009056, GO:	14.778	0.36	1.20	6.74E-04
P62829	60S ribosomal protein L23	RL23	ENSG00000125691	17	GO:0008283, GO:	14.856	1.11	2.73	3.02E-04
P62750	60S ribosomal protein L23a	RL23A	ENSG00000198242	17	GO:0008283, GO:	17.684	1.02	0.88	1.78E-04
P83731	60S ribosomal protein L24	RL24	ENSG00000114391	3	GO:0009056, GO:	17.768	1.00	1.22	5.97E-04
P61254	60S ribosomal protein L26	RL26	ENSG00000161970	17		17.248	0.44	0.14	1.67E-04
P46776	60S ribosomal protein L27a	RL27A	ENSG00000166441	11	GO:0008150, GO:	16.551	1.08	1.66	0.00E+00
P46779	60S ribosomal protein L28	RL28	ENSG00000108107	19		15.738	0.26	0.22	2.46E-04
P39023	60S ribosomal protein L3	RL3	ENSG00000100316	22	GO:0009056, GO:	46.080	0.78	0.52	3.22E-05
P62899	60S ribosomal protein L31	RL31	ENSG00000071082	2		14.454	0.12	0.46	3.90E-04
P62910	60S ribosomal protein L32	RL32	ENSG00000144713	3	GO:0008150, GO:	15.850	0.69	1.02	1.00E-04
Q9Y3U8	60S ribosomal protein L36	RL36	ENSG00000130255	19	GO:0008150, GO:	12.246	0.79	0.41	1.08E-03
P36578	60S ribosomal protein L4	RL4	ENSG00000174444	15	GO:0008150, GO:	47.667	1.55	1.75	2.26E-04

P46777	60S ribosomal protein L5	RL5	ENSG00000122406	1	GO:0009056, GO:(	34.341	1.38	0.95	1.17E-04
Q02878	60S ribosomal protein L6	RL6	ENSG00000089009	12	GO:0009056, GO:(	32.708	0.95	1.45	7.34E-05
P18124	60S ribosomal protein L7	RL7	ENSG00000147604	8	GO:0009056, GO:(	29.207	3.17	8.66	2.33E-05
P62424	60S ribosomal protein L7a	RL7A	ENSG00000280858	CHR_HG2030_P	GO:0008150, GO:(	29.977	0.94	1.15	4.15E-05
P62917	60S ribosomal protein L8	RL8	ENSG00000161016	8	GO:0008150, GO:(	28.007	0.46	0.61	3.48E-04
P05388	60S acidic ribosomal protein P0	RLA0	ENSG00000089157	12		34.252	0.24	0.39	1.45E-04
P05386	60S acidic ribosomal protein P1	RLA1	ENSG00000137818	15	GO:0008150, GO:(	11.507	0.86	1.52	6.79E-03
P05387	60S acidic ribosomal protein P2	RLA2	ENSG00000177600	11	GO:0008150, GO:(	11.658	1.73	1.55	1.57E-04
Q13151	Heterogeneous nuclear ribonucleoprotein	ROA0	ENSG00000177733	5	GO:0008150, GO:(	30.822	3.87	0.90	1.21E-04
P09651	Heterogeneous nuclear ribonucleoprotein	ROA1	ENSG00000135486	12		38.723	0.65	0.76	0.00E+00
P22626	Heterogeneous nuclear ribonucleoproteins	ROA2	ENSG00000122566	7		37.407	0.73	0.56	1.50E-04
P51991	Heterogeneous nuclear ribonucleoprotein	ROA3	ENSG00000170144	2		39.571	1.06	0.72	7.34E-05
P04843	Dolichyl-diphosphooligosaccharide--protei	RPN1	ENSG00000163902	3	GO:0008150, GO:(	68.527	1.10	0.87	9.87E-05
Q9P2E9	Ribosome-binding protein 1	RRBP1	ENSG00000125844	20	GO:0008150, GO:(	152.365	1.07	0.96	8.39E-05
P46783	40S ribosomal protein S10	RS10	ENSG00000124614	6	GO:0009056, GO:(	18.886	1.21	1.77	8.96E-04
P62280	40S ribosomal protein S11	RS11	ENSG00000142534	19	GO:0009056, GO:(	18.419	1.06	0.65	9.38E-05
P25398	40S ribosomal protein S12	RS12	ENSG00000112306	6	GO:0008150, GO:(	14.505	0.82	0.68	4.06E-05
P62277	40S ribosomal protein S13	RS13	ENSG00000110700	11	GO:0009056, GO:(	17.212	1.62	1.96	1.53E-04
P62263	40S ribosomal protein S14	RS14	ENSG00000164587	5	GO:0009056, GO:(	16.263	1.42	1.20	2.70E-04
P62841	40S ribosomal protein S15	RS15	ENSG00000115268	19	GO:0009056, GO:(	17.029	1.28	1.79	3.87E-04
P62249	40S ribosomal protein S16	RS16	ENSG00000105193	19	GO:0008150, GO:(	16.435	2.12	3.74	4.92E-05
P62269	40S ribosomal protein S18	RS18	ENSG00000226225	CHR_HSCHR6_I	GO:0009056, GO:(	17.708	0.79	2.35	1.92E-03
P39019	40S ribosomal protein S19	RS19	ENSG00000105372	19	GO:0040011, GO:(	16.051	0.69	1.14	7.80E-06
P15880	40S ribosomal protein S2	RS2	ENSG00000140988	16	GO:0009056, GO:(	31.305	1.02	1.00	1.92E-05
P63220	40S ribosomal protein S21	RS21	ENSG00000171858	20	GO:0008150, GO:(	9.106	1.29	0.76	3.06E-05
P62266	40S ribosomal protein S23	RS23	ENSG00000186468	5	GO:0008150, GO:(	15.798	1.21	0.30	1.91E-03
P62847	40S ribosomal protein S24	RS24	ENSG00000138326	10		15.413	2.73	5.33	8.15E-05
P62851	40S ribosomal protein S25	RS25	ENSG00000280831	CHR_HG2217_P	GO:0008150, GO:(	13.734	1.59	0.75	1.25E-04
P42677	40S ribosomal protein S27	RS27	ENSG00000177954	1		9.455	2.93	2.58	1.15E-05
P62857	40S ribosomal protein S28	RS28	ENSG00000233927	19	GO:0009056, GO:(	7.836	0.94	1.43	1.17E-04
P23396	40S ribosomal protein S3	RS3	ENSG00000149273	11		26.671	1.34	0.88	4.21E-04
P61247	40S ribosomal protein S3a	RS3A	ENSG00000145425	4	GO:0009056, GO:(	29.926	0.87	1.46	2.06E-04
P62701	40S ribosomal protein S4, X isoform	RS4X	ENSG00000198034	X	GO:0008283, GO:(	29.579	0.40	1.49	1.21E-04
P46782	40S ribosomal protein S5	RS5	ENSG00000083845	19	GO:0009056, GO:(	22.862	2.28	2.36	4.96E-04
P62753	40S ribosomal protein S6	RS6	ENSG00000137154	9	GO:0009056, GO:(	28.663	0.52	0.93	3.59E-05
P62081	40S ribosomal protein S7	RS7	ENSG00000171863	2	GO:0009790, GO:(	22.113	0.39	0.91	4.67E-05
P62241	40S ribosomal protein S8	RS8	ENSG00000142937	1	GO:0009056, GO:(	24.190	0.44	0.54	7.07E-04
P46781	40S ribosomal protein S9	RS9	ENSG00000278081	CHR_HSCHR19I	GO:0008283, GO:(	22.578	2.93	3.45	3.26E-04
P08865	40S ribosomal protein SA	RSSA	ENSG00000168028	3	GO:0009056, GO:(	32.833	1.10	1.21	8.52E-05
Q9Y3I0	tRNA-splicing ligase RtcB homolog	RTCB	ENSG00000100220	22	GO:0009790, GO:(	55.175	1.50	1.59	6.30E-04
P06703	Protein S100-A6	S10A6	ENSG00000197956	1	GO:0008283, GO:(	10.173	0.55	1.20	1.14E-04
P60903	Protein S100-A10	S10AA	ENSG00000197747	1	GO:0008150, GO:(	11.196	1.06	1.13	5.34E-05
P31949	Protein S100-A11	S10AB	ENSG00000163191	1	GO:0008283, GO:(	11.733	1.15	0.66	2.22E-04
Q99584	Protein S100-A13	S10AD	ENSG00000189171	1	GO:0008283, GO:(	11.464	2.90	1.20	5.30E-04

Q96FQ6	Protein S100-A16	S10AG	ENSG00000188643	1	GO:0008150, GO:	11.794	3.34	4.75	2.26E-04
P23526	Adenosylhomocysteinase	SAHH	ENSG00000101444	20	GO:0008150, GO:	47.685	0.63	0.72	3.29E-04
O75396	Vesicle-trafficking protein SEC22b	SC22B	ENSG00000265808	1	GO:0008150, GO:	24.578	1.26	0.91	1.04E-04
Q15436	Protein transport protein Sec23A	SC23A	ENSG00000100934	14		86.105	0.30	0.22	1.75E-03
O94979	Protein transport protein Sec31A	SC31A	ENSG00000138674	4		132.931	1.10	2.74	2.02E-04
Q12765	Secernin-1	SCRN1	ENSG00000136193	7		46.353	1.16	0.96	8.52E-05
P55735	Protein SEC13 homolog	SEC13	ENSG00000157020	3		35.518	0.69	0.34	1.71E-04
Q12884	Prolyl endopeptidase FAP	SEPR	ENSG00000078098	2	GO:0008283, GO:	87.657	0.39	0.96	8.93E-05
Q15019	Septin-2	SEPT2				41.461	1.05	2.15	9.07E-05
Q16181	Septin-7	SEPT7				50.648	0.75	0.29	1.34E-04
O43175	D-3-phosphoglycerate dehydrogenase	SERA	ENSG00000092621	1	GO:0009790, GO:	56.614	2.84	0.85	5.34E-05
P50454	Serpin H1	SERPH	ENSG00000149257	11	GO:0008150, GO:	46.411	0.92	1.33	2.43E-04
P0DME0	Protein SETSIP	SETLP	ENSG00000230667	1		34.861	0.48	0.40	3.70E-05
P23246	Splicing factor, proline- and glutamine-rich	SFPQ	ENSG00000116560	1	GO:0006464, GO:	76.102	0.77	0.85	4.73E-04
O75368	SH3 domain-binding glutamic acid-rich-like	SH3L1	ENSG00000131171	X	GO:0005634, GO:	12.766	0.52	0.43	2.60E-04
Q9H299	SH3 domain-binding glutamic acid-rich-like	SH3L3	ENSG00000142669	1	GO:0008150, GO:	10.431	3.79	3.04	1.43E-04
P63208	S-phase kinase-associated protein 1	SKP1	ENSG00000113558	5	GO:0006461, GO:	18.646	0.40	0.40	2.70E-04
Q7KZF4	Staphylococcal nuclease domain-containin	SND1	ENSG00000197157	7	GO:0008150, GO:	101.934	1.80	1.01	1.88E-04
P04179	Superoxide dismutase [Mn], mitochondrial	SODM	ENSG00000112096	6		24.735	1.09	1.31	3.18E-05
Q01082	Spectrin beta chain, non-erythrocytic 1	SPTB2	ENSG00000115306	2	GO:0007010, GO:	274.439	1.92	0.67	2.13E-04
Q13813	Spectrin alpha chain, non-erythrocytic 1	SPTN1	ENSG00000197694	9		284.364	1.50	0.71	1.28E-05
Q14247	Src substrate cortactin	SRC8	ENSG00000085733	11		61.549	1.62	0.33	2.60E-04
Q01130	Serine/arginine-rich splicing factor 2	SRSF2	ENSG00000161547	17		25.461	0.82	0.84	2.14E-05
P84103	Serine/arginine-rich splicing factor 3	SRSF3	ENSG00000112081	6		19.318	0.41	0.71	3.46E-05
Q04837	Single-stranded DNA-binding protein, mito	SSBP	ENSG00000262771	CHR_HSCHR7_1	GO:0008150, GO:	17.249	0.68	1.14	1.38E-04
P43307	Translocon-associated protein subunit alp	SSRA	ENSG00000124783	6	GO:0008283, GO:	32.215	2.81	3.24	1.57E-04
P42224	Signal transducer and activator of transcri	STAT1	ENSG00000115415	2	GO:0008283, GO:	87.280	1.32	1.84	4.11E-04
P31948	Stress-induced-phosphoprotein 1	STIP1	ENSG00000168439	11		62.599	0.36	0.76	9.38E-05
P16949	Stathmin	STMN1	ENSG00000117632	1		17.292	1.10	1.06	1.03E-03
Q86Y82	Syntaxin-12	STX12	ENSG00000117758	1	GO:0061024, GO:	31.622	0.36	0.30	1.10E-04
O15400	Syntaxin-7	STX7	ENSG00000079950	6		29.797	0.97	0.80	2.19E-04
O43776	Asparagine--tRNA ligase, cytoplasmic	SYNC	ENSG00000134440	18	GO:0008150, GO:	62.903	1.45	1.32	7.49E-05
P26639	Threonine--tRNA ligase, cytoplasmic	SYTC	ENSG00000113407	5		83.382	1.44	2.71	1.78E-05
P23381	Tryptophan--tRNA ligase, cytoplasmic	SYWC	ENSG00000140105	14	GO:0008283, GO:	53.132	1.01	0.66	3.44E-04
Q01995	Transgelin	TAGL	ENSG00000149591	11	GO:0008150, GO:	22.596	3.02	3.85	4.92E-05
P37802	Transgelin-2	TAGL2	ENSG00000158710	1		22.377	2.44	1.23	3.59E-05
P37837	Transaldolase	TALDO	ENSG00000177156	11	GO:0008150, GO:	37.516	0.81	1.08	1.39E-04
P0DPH7	Tubulin alpha-3C chain	TBA3C	ENSG00000198033	13		49.928	1.37	0.56	1.10E-04
Q13885	Tubulin beta-2A chain	TBB2A	ENSG00000137267	6		49.875	0.99	0.83	9.87E-05
P68371	Tubulin beta-4B chain	TBB4B	ENSG00000188229	9	GO:0007010, GO:	49.799	0.84	0.74	1.79E-04
P07437	Tubulin beta chain	TBB5	ENSG00000232575	CHR_HSCHR6_I	GO:0007010, GO:	49.639	1.21	1.25	1.25E-04
Q9BUF5	Tubulin beta-6 chain	TBB6	ENSG00000176014	18	GO:0008150, GO:	49.825	0.72	0.89	2.20E-04
P53999	Activated RNA polymerase II transcription	TCP4	ENSG00000113387	5	GO:0008150, GO:	14.386	1.14	0.43	1.61E-04
P17987	T-complex protein 1 subunit alpha	TCPA	ENSG00000120438	6	GO:0006461, GO:	60.306	1.93	5.72	2.83E-04

P78371	T-complex protein 1 subunit beta	TCPB	ENSG00000166226	12		57.452	0.76	1.17	1.37E-04
P50991	T-complex protein 1 subunit delta	TCPD	ENSG00000115484	2		57.888	2.31	3.33	3.11E-05
P48643	T-complex protein 1 subunit epsilon	TCPE	ENSG00000150753	5	GO:0043167, GO:	59.633	1.80	3.59	7.81E-05
P49368	T-complex protein 1 subunit gamma	TCPG	ENSG00000163468	1	GO:0006461, GO:	60.495	1.30	1.01	6.01E-04
Q99832	T-complex protein 1 subunit eta	TCPH	ENSG00000135624	2	GO:0008150, GO:	59.329	1.13	1.13	1.16E-05
P50990	T-complex protein 1 subunit theta	TCPQ	ENSG00000156261	21	GO:0016887, GO:	59.583	0.73	0.99	4.15E-05
P40227	T-complex protein 1 subunit zeta	TCPZ	ENSG00000146731	7		57.988	0.90	1.83	4.45E-05
P13693	Translationally-controlled tumor protein	TCTP	ENSG00000133112	13	GO:0043167, GO:	19.583	0.75	1.17	1.07E-04
Q15185	Prostaglandin E synthase 3	TEBP	ENSG00000110958	12		18.685	0.35	0.87	1.50E-04
P55072	Transitional endoplasmic reticulum ATPas	TERA	ENSG00000165280	9	GO:0016887, GO:	89.266	1.16	1.22	1.62E-05
P10599	Thioredoxin	THIO	ENSG00000136810	9		11.730	0.54	0.81	8.15E-05
Q13263	Transcription intermediary factor 1-beta	TIF1B	ENSG00000130726	19		88.493	2.90	2.39	2.87E-05
P29401	Transketolase	TKT	ENSG00000163931	3		67.835	0.63	0.34	1.19E-05
Q9Y490	Talin-1	TLN1	ENSG00000137076	9	GO:0007010, GO:	269.599	0.84	0.85	1.64E-04
P49755	Transmembrane emp24 domain-containin	TMEDA	ENSG00000170348	14	GO:0008150, GO:	24.960	2.00	0.75	1.71E-04
O60784	Target of Myb protein 1	TOM1	ENSG00000100284	22		53.785	1.27	0.22	2.55E-04
P60174	Triosephosphate isomerase	TPIS	ENSG00000111669	12		30.772	0.61	0.56	6.93E-05
P07951	Tropomyosin beta chain	TPM2	ENSG00000198467	9		32.831	1.89	1.31	8.71E-04
P06753	Tropomyosin alpha-3 chain	TPM3	ENSG00000143549	1		32.930	2.16	1.28	6.30E-04
P67936	Tropomyosin alpha-4 chain	TPM4	ENSG00000167460	19	GO:0008150, GO:	28.504	1.33	0.51	1.58E-05
Q16881	Thioredoxin reductase 1, cytoplasmic	TRXR1	ENSG00000198431	12	GO:0008150, GO:	70.862	2.76	3.86	4.96E-05
P07996	Thrombospondin-1	TSP1	ENSG00000137801	15	GO:0008283, GO:	129.300	3.95	6.58	3.40E-05
Q8NBS9	Thioredoxin domain-containing protein 5	TXND5	ENSG00000239264	6		47.599	0.88	1.74	1.95E-03
P63313	Thymosin beta-10	TYB10	ENSG00000034510	2	GO:0005623, GO:	5.023	0.82	0.46	5.01E-04
P62328	Thymosin beta-4	TYB4	ENSG00000205542	X	GO:0007010, GO:	5.050	0.63	0.38	1.50E-04
P22314	Ubiquitin-like modifier-activating enzyme 1	UBA1	ENSG00000130985	X		117.774	0.97	1.13	1.85E-04
P0CG47	Polyubiquitin-B	UBB	ENSG00000170315	17		25.746	1.02	0.73	4.79E-05
P61088	Ubiquitin-conjugating enzyme E2 N	UBE2N	ENSG00000177889	12	GO:0043167, GO:	17.127	1.83	1.59	4.56E-05
P09936	Ubiquitin carboxyl-terminal hydrolase isozy	UCHL1	ENSG00000154277	4	GO:0008283, GO:	24.808	1.50	1.11	1.54E-03
O60701	UDP-glucose 6-dehydrogenase	UGDH	ENSG00000109814	4		54.989	0.40	5.54	5.10E-04
Q9NYU2	UDP-glucose:glycoprotein glucosyltransfer	UGGG1	ENSG00000136731	2		177.078	1.00	0.34	7.18E-04
Q16851	UTP--glucose-1-phosphate uridylyltransfer	UGPA	ENSG00000169764	2		56.905	0.68	0.75	2.78E-05
Q15836	Vesicle-associated membrane protein 3	VAMP3	ENSG00000049245	1	GO:0006461, GO:	11.302	1.11	1.02	1.64E-05
Q99536	Synaptic vesicle membrane protein VAT-1	VAT1	ENSG00000108828	17	GO:0008150, GO:	41.893	1.40	1.15	9.87E-05
Q00341	Vigilin	VIGLN	ENSG00000115677	2		141.368	0.87	0.64	1.50E-05
P08670	Vimentin	VIME	ENSG00000026025	10	GO:0008150, GO:	53.619	0.98	2.61	2.75E-05
P18206	Vinculin	VINC	ENSG00000035403	10		123.722	1.18	1.09	1.88E-05
O75436	Vacuolar protein sorting-associated protei	VP26A	ENSG00000122958	10	GO:0008150, GO:	38.146	1.29	0.80	6.24E-05
O75083	WD repeat-containing protein 1	WDR1	ENSG00000071127	4	GO:0007010, GO:	66.152	1.59	1.44	5.50E-05
P13010	X-ray repair cross-complementing protein	XRCC5	ENSG00000079246	2	GO:0016887, GO:	82.652	1.13	1.01	2.28E-05
P12956	X-ray repair cross-complementing protein	XRCC6	ENSG00000196419	22		69.799	0.88	1.07	1.06E-04
P67809	Nuclease-sensitive element-binding protei	YBOX1	ENSG00000065978	1	GO:0009790, GO:	35.903	1.82	0.86	1.71E-04
Q15942	Zyxin	ZYX	ENSG00000159840	7	GO:0008150, GO:	61.238	1.17	0.97	1.10E-04
